# Targeting the NLRP3 inflammasome in rheumatoid arthritis fibroblast-like synoviocytes: pathogenic mechanisms, therapeutic strategies, and translational perspectives

**DOI:** 10.3389/fphar.2026.1955904

**Published:** 2026-09-29

**Authors:** Lunmin Bao, Haibing Dai, Daoming Wang, Hongmei Jiang

**Affiliations:** 1 Department of Laboratory Medicine, People’s Hospital of Anshun City, Anshun, China; 2 School of Clinical Laboratory Science, Guizhou Medical University, Guiyang, China; 3 Department of Medical Technology, Zunyi Medical and Pharmaceutical College, Zunyi, China

**Keywords:** clinical translation, fibroblast-like synoviocytes, nanomedicine, NLRP3 inflammasome, rheumatoid arthritis, targeted therapy

## Abstract

Rheumatoid arthritis (RA) is a chronic systemic autoimmune disease characterized by persistent synovitis, pannus formation, cartilage degradation, and bone erosion. Although conventional synthetic, biologic, and targeted synthetic disease-modifying antirheumatic drugs have improved disease control, many patients fail to achieve sustained remission, lose responsiveness over time, or develop treatment-limiting safety concerns. Fibroblast-like synoviocytes (FLSs) are increasingly recognized as long-lived pathogenic stromal cells that sustain synovial inflammation, promote matrix degradation, support osteoclastogenesis, and shape immune and vascular crosstalk within the rheumatoid joint. The NOD-like receptor family pyrin domain-containing 3 (NLRP3) inflammasome provides a mechanistic link between synovial stress responses and inflammatory amplification. In RA-FLSs, defined inflammatory, hypoxic and oxidative-stress conditions can induce NLRP3-related signaling, whereas other proposed triggers, including lysosomal injury, extracellular ATP and ion flux, are supported more strongly by broader synovial or general inflammasome evidence. Through caspase-1 activation, interleukin (IL)-1β and IL-18 maturation, and gasdermin D-mediated pyroptotic signaling, NLRP3 can reinforce stromal inflammation and tissue-destructive synovial remodeling. In this review, we summarize canonical, non-canonical, and alternative mechanisms of NLRP3 inflammasome activation, with emphasis on their relevance to RA-FLS biology and the inflamed synovial niche. We discuss how NLRP3 signaling intersects with fibroblast heterogeneity, macrophage–FLS crosstalk, endothelial activation, chondrocyte injury, osteoclastogenesis, and context-dependent pyroptosis. We further evaluate pharmacological strategies targeting the NLRP3 axis, including direct NLRP3 inhibitors, upstream stress modulators, caspase-1 or gasdermin D blockade, IL-1β/IL-18 pathway inhibition, natural products, repurposed molecules, nanomedicine-based delivery, and rational combination regimens. Key translational issues include FLS specificity, synovial drug exposure, target engagement, chronic safety, biomarker-guided patient selection, and mechanism-aligned endpoints. An RA-FLS-centered view of NLRP3 targeting may help refine anti-inflammatory pharmacology and support the development of synovium-directed therapeutic strategies for selected patients with difficult-to-treat RA.

## 1 Introduction

Rheumatoid arthritis (RA) remains a highly disabling autoimmune rheumatic disease in which persistent inflammation leads to irreversible articular damage, functional impairment, and systemic comorbidities, with an estimated prevalence approaching 1% worldwide (Smolen et al., 2016; Almutairi et al., 2021; Klingemann et al., 2026). Since the approval of low-dose methotrexate, the therapeutic armamentarium has expanded to include conventional synthetic disease-modifying antirheumatic drugs (csDMARDs), biological DMARDs (bDMARDs), and targeted synthetic DMARDs (tsDMARDs) (Smolen et al., 2023). Current American College of Rheumatology (ACR) and European Alliance of Associations for Rheumatology (EULAR) recommendations emphasize treat-to-target management, sustained control of disease activity, and individualized escalation of therapy (Fraenkel et al., 2021; Smolen et al., 2023). Despite these advances, a substantial subset of patients does not achieve durable remission, develops secondary loss of response, or remains unsuitable for prolonged treatment because of infection risk, hepatic or hematologic toxicity, cardiovascular safety concerns, malignancy-related caution, comorbidity burden, or economic constraints (Nagy et al., 2022). These limitations have shifted attention from single-cytokine blockade alone toward the synovial tissue circuits that sustain inflammation even when systemic immune activation is partially suppressed.

Fibroblast-like synoviocytes (FLSs) are central effector cells within the pathological synovial network. Under physiological conditions, FLSs contribute to joint lubrication, extracellular matrix (ECM) homeostasis, and tissue repair (Mahmoud et al., 2022). In RA, FLSs acquire an aggressive, semi-autonomous phenotype characterized by aberrant proliferation, apoptosis resistance, enhanced migration and invasion, and excessive secretion of IL-6, granulocyte-macrophage colony-stimulating factor, chemokines, matrix metalloproteinases (MMPs), and receptor activator of nuclear factor-κB ligand (RANKL) (Karami et al., 2020; Mousavi et al., 2021; Qian et al., 2024; Yue et al., 2025). Human single-cell and spatial transcriptomic studies have further shown that the RA synovium is a heterogeneous inflammatory tissue rather than a uniform lesion (Smith et al., 2023). Sublining fibroblast subsets are closely associated with cytokine and chemokine networks, whereas lining layer fibroblasts predominantly mediate cartilage invasion and tissue damage (Mizoguchi et al., 2018; Croft et al., 2019; Zhang et al., 2019). Together, these observations support FLSs as attractive therapeutic targets, provided that pathological activation states can be selectively suppressed without compromising fibroblast populations required for normal tissue integrity.

The NOD-like receptor family pyrin domain-containing 3 (NLRP3) inflammasome is a cytosolic stress-sensing platform that converts endogenous and exogenous danger signals into caspase-1 activation, IL-1β and IL-18 maturation, and gasdermin D (GSDMD)-mediated pyroptotic cell death (Lamkanfi and Dixit, 2014; Shi et al., 2015; He et al., 2016a; Swanson et al., 2019). NLRP3 responds to potassium efflux, mitochondrial dysfunction, lysosomal injury, reactive oxygen species (ROS), metabolic stress, extracellular ATP, and related perturbations, making it well suited to sense the abnormal microenvironment of the rheumatoid joint (Koumangoye, 2022; Shin et al., 2026). The RA synovium is enriched in pro-inflammatory cytokines, immune complexes, damage-associated molecular patterns (DAMPs), hypoxia, lactate, lipid mediators, and fragmented matrix components (Mao et al., 2023; Zhang X. et al., 2023). These cues can provide two functionally distinct signals for inflammasome activation: a priming signal that upregulates NLRP3 and inflammatory substrates, and an activation signal that promotes inflammasome assembly.

A focus on NLRP3 signaling in FLSs is therefore biologically and translationally justified. The NLRP3/caspase-1/IL-1β/IL-18/GSDMD cascade contributes to synovial inflammation, bone erosion, and extra-articular inflammatory burden in RA (Zhou et al., 2023; Chen P. K. et al., 2024; Li et al., 2025). Drug discovery has moved from broad anti-inflammatory intervention toward selective NLRP3 inhibition, with candidate compounds such as dapansutrile/OLT1177, selnoflast, DFV890, and NT-0796 progressing through preclinical or clinical development in inflammatory disorders (Sánchez-Fernández et al., 2019; Harrison et al., 2023; Klughammer et al., 2023; Gatlik et al., 2024). In parallel, nanomedicine, biomimetic carriers, and ligand-directed delivery strategies may increase synovial enrichment of NLRP3 modulators and reduce systemic exposure (Thomas et al., 2011; Li J. et al., 2024; Hu et al., 2026). This review summarizes the biological basis of NLRP3 inflammasome activation, the pathogenic phenotype of RA-FLSs, the therapeutic landscape of NLRP3 inhibitors, and the translational requirements for clinical development. The central premise is that NLRP3-targeted therapy in RA will be most credible when coupled to biomarker stratification, synovial microenvironment targeting, and rational combination strategies, rather than pursued as non-selective systemic inflammasome suppression.

## 2 Biological basis of NLRP3 inflammasome activation

### 2.1 NLRP3 structure and canonical two-signal activation

Inflammasomes are multimeric protein complexes that sense infection, tissue injury, and disruption of cellular homeostasis. Unlike inflammasome sensors that recognize defined microbial ligands, NLRP3 responds to a broad spectrum of cellular stressors, including extracellular ATP, pore-forming toxins, crystalline material, mitochondrial injury, lysosomal rupture, and metabolic stress (Xu and Núñez, 2023). NLRP3 contains an N-terminal pyrin domain, a central nucleotide-binding and oligomerization (NACHT) domain, and C-terminal leucine-rich repeats. Canonical activation is commonly described as a two-signal process. The priming signal (signal 1), typically mediated by Toll-like receptor (TLR), TNF receptor, or IL-1 receptor signaling, activates nuclear factor-κB (NF-κB) and induces NLRP3, pro-IL-1β, and related inflammatory genes. The activation signal (signal 2) promotes conformational remodeling, oligomerization, recruitment of the adaptor apoptosis-associated speck-like protein containing a CARD (ASC), and proximity-induced caspase-1 activation (Xu and Núñez, 2023).

Activated caspase-1 converts pro-IL-1β and pro-IL-18 into mature cytokines and cleaves GSDMD to generate its N-terminal pore-forming fragment. GSDMD pores facilitate cytokine release and, when formed extensively, induce pyroptotic membrane permeabilization and cell lysis (He et al., 2015; Kayagaki et al., 2015; Shi et al., 2015; He et al., 2016a). Pyroptosis is immunologically distinct from apoptosis, which generally preserves membrane integrity and is comparatively non-inflammatory, whereas pyroptosis releases cytokines, alarmins, lactate dehydrogenase, and intracellular DAMPs that amplify local inflammation. This distinction is therapeutically relevant in RA, where synovial hyperplasia and tissue destruction involve both survival of pathogenic FLSs and inflammatory mediator release. The consequences of pyroptosis modulation are therefore context dependent; suppression of pyroptosis may reduce inflammatory amplification but could also alter clearance of activated stromal cells.

### 2.2 Upstream regulatory nodes and pharmacological implications

NLRP3 activation is controlled by multiple upstream regulatory cascades. Potassium efflux is a widely observed proximal event downstream of ATP-P2X7 signaling and membrane damage (Koumangoye, 2022). Mitochondrial ROS generation and mitochondrial DNA release are important upstream mechanisms of NLRP3 activation (Nakahira et al., 2011; Zhou et al., 2011). Ion flux and lysosomal disruption provide additional activation signals in appropriate cellular contexts. In addition, diverse NLRP3 agonists induce trans-Golgi network disassembly, with NLRP3 subsequently recruited to PtdIns4P-enriched dispersed trans-Golgi network structures that support inflammasome assembly (Chen and Chen, 2018). NEK7 is an essential licensing factor for NLRP3 activation downstream of potassium efflux and participates in assembly of the active inflammasome complex (He et al., 2016b; Sharif et al., 2019; Andreeva et al., 2021). These inputs converge on the NACHT domain, which binds and hydrolyzes ATP to support oligomerization. This structural insight is highly relevant to pharmacological intervention. Direct NLRP3 inhibitors typically act by stabilizing inactive conformations, interfering with ATPase activity, or disrupting inflammasome assembly, rather than by broadly suppressing upstream inflammatory signaling. (Coll et al., 2015; Coll et al., 2019; Tapia-Abellán et al., 2019; Vande Walle and Lamkanfi, 2024).

### 2.3 Non-canonical pathways and RA-specific regulatory context

Non-canonical and alternative inflammasome pathways expand the biological scope of NLRP3 signaling beyond the classical macrophage model. In humans, caspase-4 and caspase-5 detect cytosolic lipopolysaccharide, whereas murine caspase-11 mediates an analogous pathway. These inflammatory caspases cleave GSDMD and can secondarily activate NLRP3 through potassium efflux. Alternative NLRP3 activation can promote IL-1β release with less prominent pyroptotic lysis, indicating that inflammasome activation does not necessarily produce identical cellular outcomes across cell types (Gaidt et al., 2016). The canonical two-signal pathway, cytosolic LPS-driven non-canonical pathway, and single-signal alternative NLRP3 activation pathway are summarized in Figure 1. The RA synovium contains macrophages, monocytes, neutrophils, dendritic cells, and stromal cells that may engage overlapping inflammasome-related pathways. Accordingly, intrinsic NLRP3 signaling in FLSs operates within a broader synovial inflammasome network and is unlikely to function as an autonomous molecular switch.

**FIGURE 1:**
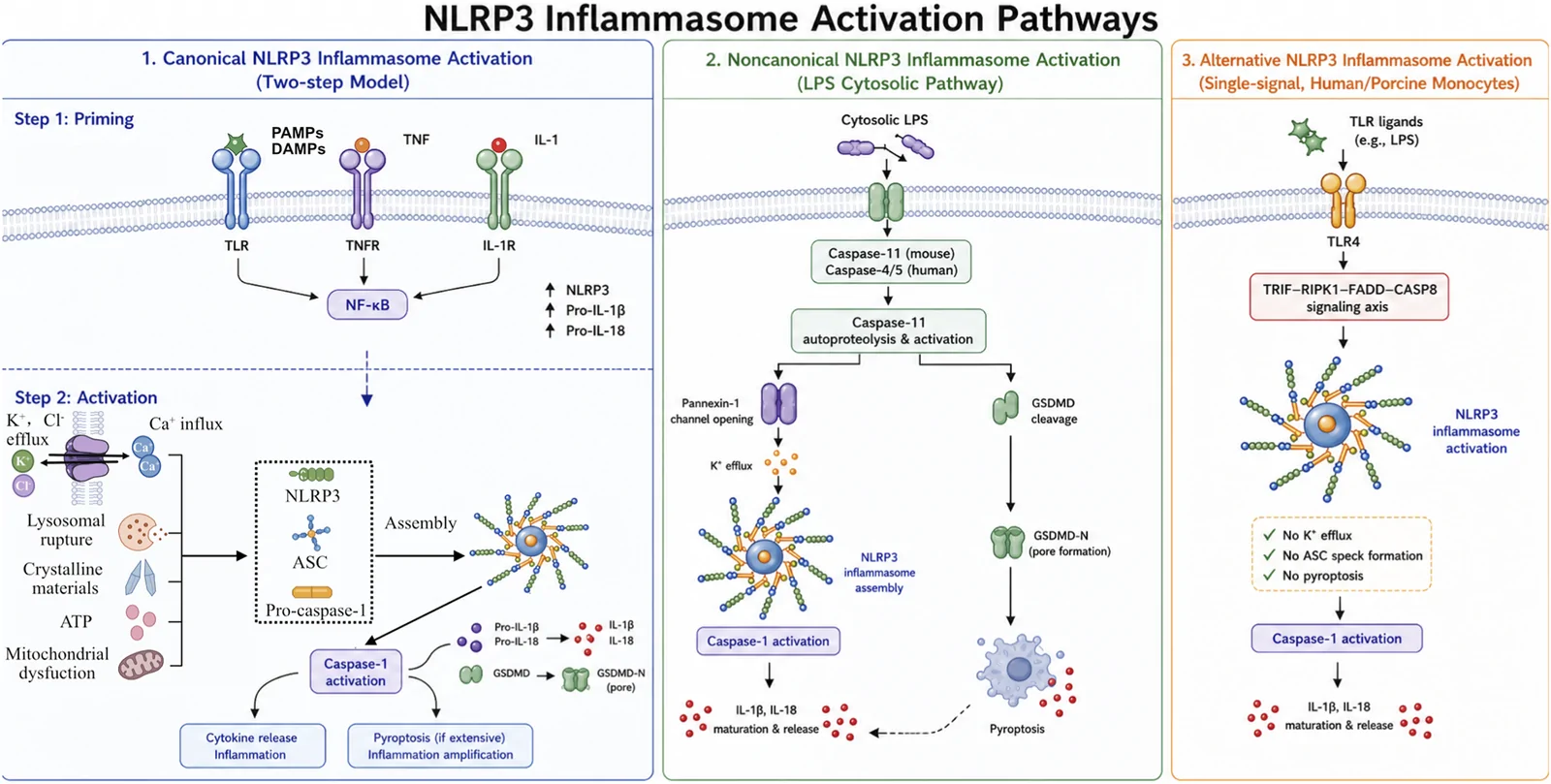
Major pathways of NLRP3 inflammasome activation. Canonical NLRP3 activation is commonly initiated by a priming signal mediated by Toll-like receptor (TLR), tumor necrosis factor receptor (TNFR), or interleukin-1 receptor (IL-1R) signaling, which activates NF-κB and upregulates NLRP3, pro-IL-1β, and pro-IL-18. A second activation signal, such as extracellular ATP, potassium efflux, chloride flux, calcium mobilization, lysosomal rupture, crystalline materials, or mitochondrial dysfunction, promotes NLRP3 conformational remodeling, ASC recruitment, inflammasome assembly, and caspase-1 activation. Activated caspase-1 processes pro-IL-1β and pro-IL-18 into mature IL-1β and IL-18 and cleaves gasdermin D (GSDMD), leading to cytokine release and, when extensive, pyroptotic cell death. In the non-canonical pathway, cytosolic lipopolysaccharide (LPS) activates inflammatory caspases, including caspase-4/5 in humans and caspase-11 in mice, which cleave GSDMD and can secondarily activate NLRP3 through potassium efflux. Alternative NLRP3 activation, described mainly in human monocytes, can induce caspase-1 activation and IL-1β release with limited potassium efflux, ASC speck formation, and pyroptosis. The schematic illustration was created using Affinity Designer.

Priming represents a disease-modifiable regulatory node that actively sets the threshold for subsequent inflammasome activation. In RA synovium, NF-κB activation is driven by tumor necrosis factor-α (TNF-α), IL-1β, immune complexes, TLR ligands, and mechanical injury, whereas JAK/STAT signaling provides additional cytokine-driven amplification. These pathways regulate not only NLRP3 transcription but also pro-IL-1β, pro-IL-18, purinergic receptors, metabolic enzymes, and antioxidant programs, thereby reshaping the threshold for inflammasome activation in response to secondary stress signals (Yin et al., 2022). In FLSs exposed to sustained cytokine pressure rather than acute microbial challenge, priming may be chronic and spatially restricted to pannus, perivascular regions, or cartilage-invasive interfaces. This spatial dependence may explain why tissue-level inflammasome inhibition can be biologically meaningful even when systemic inflammatory markers are only moderately elevated.

Post-translational regulation adds further complexity to NLRP3 biology. Phosphorylation, ubiquitination, deubiquitination, sumoylation, chaperone interactions, and organelle membrane associations collectively regulate the transition of NLRP3 among inactive, licensed, and assembly-competent states (O'Keefe et al., 2024). These regulatory layers provide multiple pharmacologically actionable nodes, including ATPase activity, NEK7 binding, ASC recruitment, conformational stabilization, and upstream organelle stress. In RA, an ideal inhibitor would suppress disease-relevant inflammasome activation under chronic inflammatory conditions without abolishing protective innate immune functions.

## 3 Pathogenic phenotype of RA-FLSs

### 3.1 Pathogenic effector functions and molecular imprinting

FLSs occupy an anatomically and functionally central position at the interface between immune activation and tissue destruction. They respond to cytokines derived from macrophages, T cells, B cells, mast cells, and neutrophils, while actively shaping the synovial inflammatory microenvironment through chemokine gradients, adhesion molecules, and ECM remodeling (Noss and Brenner, 2008; Buckley et al., 2021). Activated FLSs secrete IL-6, IL-8, CCL2, CXCL12, CXCL13, and granulocyte-macrophage colony-stimulating factor (GM-CSF), thereby promoting immune cell recruitment and retention within the joint (Mizoguchi et al., 2018). They also produce MMP1, MMP3, MMP9, and MMP13, which contribute to cartilage degradation, and express RANKL, which supports osteoclast differentiation and bone erosion (Croft et al., 2019). RA synovial fibroblasts can migrate and mediate cartilage destruction at distant sites in experimental models (Lefèvre et al., 2009). More recent studies support the concept that pathogenic fibroblast states can become stably maintained through transcriptional and epigenetic remodeling (Croft et al., 2019; Qian et al., 2024).

The RA-FLS phenotype is maintained by interconnected molecular programs. Inflammatory priming by TNF-α, IL-1β, IL-17, and TLR ligands activates NF-κB, activator protein-1 (AP-1), mitogen-activated protein kinase (MAPK), and JAK/STAT signaling. Epigenetic alterations, including DNA methylation, histone modification, and noncoding RNA regulation, further stabilize pathogenic gene expression programs (Karami et al., 2020; Qian et al., 2024). Metabolic reprogramming, particularly enhanced glycolysis, mitochondrial dysfunction, and altered lipid metabolism, supports proliferation, cytokine output, and invasiveness (Falconer et al., 2018; Fearon et al., 2019). These pathways are not independent modules; instead, inflammatory signaling modifies metabolism, metabolic intermediates influence chromatin and transcription, and mitochondrial stress can lower the threshold for inflammasome activation.

### 3.2 Fibroblast heterogeneity and therapeutic implications

Single-cell and spatial transcriptomic technologies have refined the understanding of RA-FLS heterogeneity. THY1-negative lining-like fibroblasts, often marked by PRG4, CLIC5, and TSPAN15, are associated at the transcriptomic level with matrix-remodeling and cartilage- or bone-erosive programs, whereas THY1-positive sublining fibroblasts are enriched for cytokine and chemokine expression and contribute to leukocyte recruitment and inflammatory organization (Mizoguchi et al., 2018; Croft et al., 2019). These subsets represent functionally specialized pathogenic states rather than descriptive categories. Inflammatory FLSs can support leukocyte recruitment and retention and participate in reciprocal inflammatory crosstalk with immune cells (Buckley et al., 2021; Smith et al., 2023). Invasive lining FLSs, by contrast, are enriched at the cartilage-pannus junction and contribute directly to cartilage destruction. This compartmentalization is important for NLRP3 biology because priming and activation signals may differ between inflammatory sublining niches and mechanically stressed invasive lining regions.

Therapeutic targeting of FLSs differs fundamentally from strategies directed against macrophages or lymphocytes. Macrophages are professional inflammasome-responsive cells, whereas FLSs are long-lived stromal cells embedded within the synovial tissue architecture. Even a relatively modest FLS-intrinsic inflammatory program may exert substantial cumulative effects because FLSs persist in the joint, resist apoptosis, and provide a structural scaffold for chronic synovitis. This persistence makes FLSs attractive targets for disease modification, particularly in patients with residual synovitis despite biologic cytokine blockade. However, it also underscores the importance of cell-type and state-specific intervention. Broad suppression of fibroblast repair functions could impair tissue maintenance, whereas selective attenuation of pathogenic inflammasome activity may recalibrate the local inflammatory milieu without inducing global stromal depletion.

## 4 NLRP3 signaling in RA-FLSs and the inflamed synovial niche

Multiple inflammatory and stress-related cues within the rheumatoid synovium may contribute to NLRP3 activation, although their direct validation in RA-FLSs is uneven. Defined inflammatory, hypoxic and oxidative-stress conditions have been demonstrated to induce NLRP3-related signaling in FLS-based RA models, whereas the roles of DAMPs, lysosomal injury, extracellular ATP and ion flux are supported more strongly by myeloid, whole-synovium or general inflammasome studies. Once activated under appropriate conditions, FLS-intrinsic NLRP3 signaling can promote caspase-1 activation, IL-1β/IL-18 maturation and GSDMD-related pyroptotic signaling and may thereby contribute to broader synovial crosstalk. Once activated, the NLRP3 inflammasome promotes caspase-1 activation, IL-1β/IL-18 maturation, and GSDMD-mediated pyroptotic signaling, thereby amplifying synovial crosstalk with macrophages, T cells, chondrocytes, endothelial cells, and osteoclast-lineage cells (Figure 2).

**FIGURE 2:**
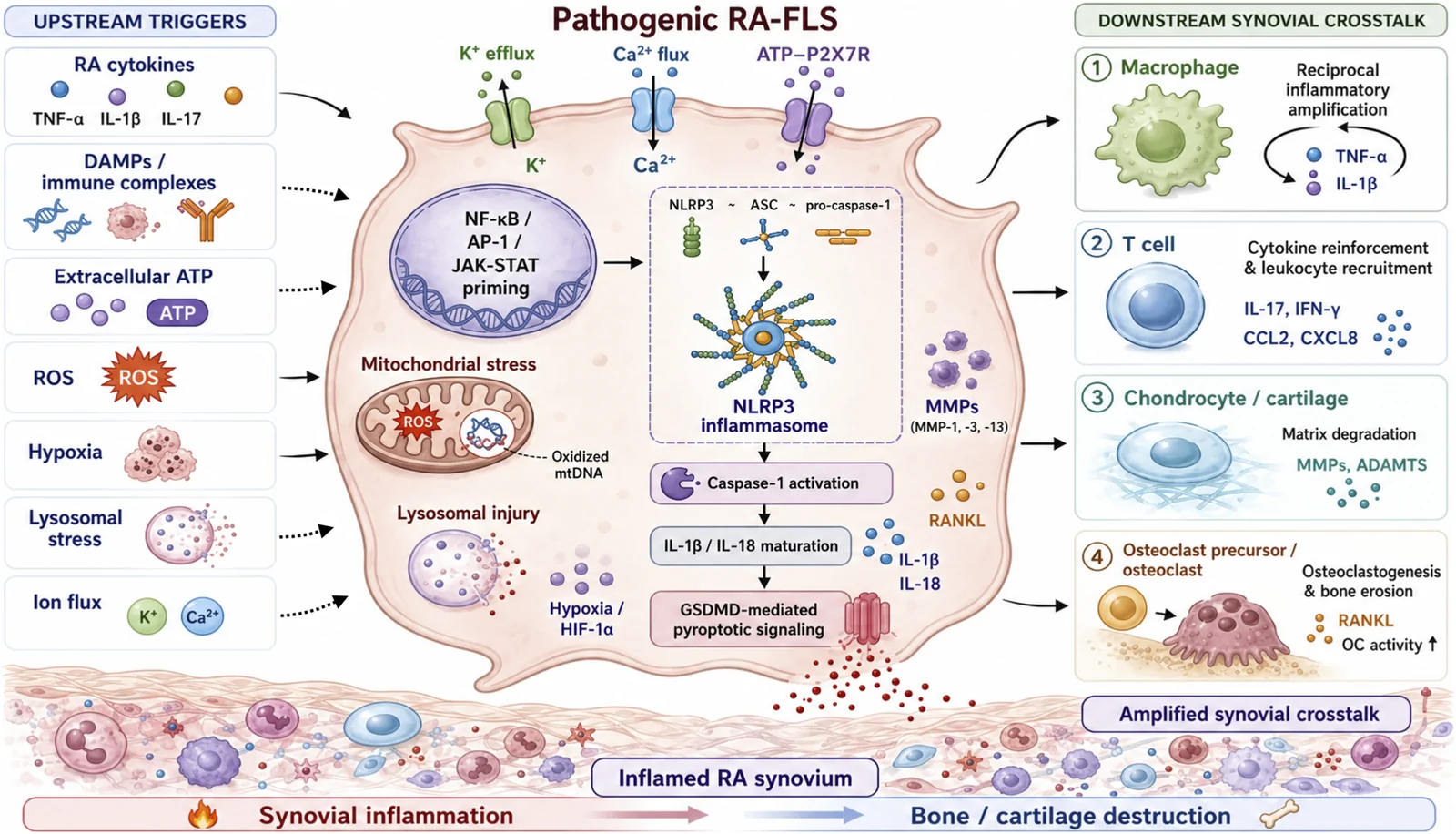
Evidence-resolved model of FLS-associated NLRP3 signaling in RA synovium. The schematic distinguishes mechanisms directly supported in RA-FLS experimental systems from pathways inferred from bulk RA synovium, myeloid cells, animal models, or general inflammasome biology. Solid arrows indicate mechanisms with direct or relatively strong RA-FLS support, whereas dashed arrows indicate indirect, context-dependent, or extrapolated relationships. Defined inflammatory, hypoxic and oxidative-stress conditions can induce NLRP3-related signaling in RA-FLSs, while the roles of DAMPs, extracellular ATP/P2X7 signaling, lysosomal injury and ion flux are less completely established in the FLS compartment. Downstream interactions with macrophages, T cells, chondrocytes and osteoclast-lineage cells represent broader synovial network effects. Importantly, studies of intact RA synovium indicate that macrophages may constitute a major inflammasome-active compartment; the figure therefore does not imply that FLSs are the predominant source of NLRP3 activity in all RA synovia. The schematic illustration was created using Affinity Designer.

### 4.1 Synovial priming and stress signals driving NLRP3 activation

The RA joint provides a highly permissive microenvironment for NLRP3 priming. TNF-α and related inflammatory signals can prime NLRP3-associated inflammatory programs in RA-FLSs under defined experimental conditions. Direct FLS-based studies have demonstrated induction of NLRP3-related components and functional inflammasome activation in response to appropriate priming and activation signals (Liu et al., 2019; Hong et al., 2022; Luo et al., 2022). Other inflammatory inputs, including IL-17 and TLR-associated signals, may further reinforce synovial priming, although their direct contribution to FLS-intrinsic NLRP3 activation remains less completely defined (Yin et al., 2022). Accordingly, NLRP3 activation in FLSs is unlikely to result from a single isolated stimulus. General inflammasome biology supports a threshold-dependent model in which multiple priming and activation signals accumulate to permit NLRP3 assembly, although direct validation of each individual input in RA-FLSs remains incomplete (Kelley et al., 2019).

Beyond these generic inflammatory and stress signals, anti-citrullinated protein antibodies (ACPAs) provide an RA-specific link between adaptive autoimmunity and NLRP3 inflammasome activation. Dong et al. found higher synovial IL-1β expression in ACPA-positive than in ACPA-negative patients with RA and showed that ACPAs purified from patients with RA promoted NLRP3-dependent IL-1β production in PBMC-derived macrophages. Mechanistically, ACPAs interacted with CD147 and enhanced CD147–integrin β1 signaling, leading to Akt/NF-κB activation and increased NLRP3 and pro-IL-1β expression. In parallel, ACPA stimulation activated pannexin channels and promoted extracellular ATP release, which engaged P2X7 and provided an activation signal for the NLRP3 inflammasome (Dong et al., 2020). These findings identify ACPAs as a disease-relevant upstream trigger capable of coupling RA-specific autoimmunity to inflammasome activation. Importantly, however, the direct mechanistic evidence in this study was obtained predominantly in monocyte-derived macrophages rather than RA-FLSs. Whether ACPAs directly induce comparable NLRP3 activation in FLSs, or instead influence the stromal compartment indirectly through macrophage-derived IL-1β and other inflammatory mediators, remains to be determined.

Mitochondrial dysfunction represents a major activation route within this framework. RA-FLSs reside in an inflamed, hypoxic, and metabolically heterogeneous microenvironment. Enhanced glycolysis supports proliferation and cytokine production; however, altered mitochondrial respiration can promote excessive ROS generation, mitochondrial DNA oxidation, and release of mitochondrial DAMPs (Biniecka et al., 2011; Bustamante et al., 2017; Weyand and Goronzy, 2017; Li P. et al., 2024). Defective mitophagy further permits accumulation of damaged mitochondria. In this context, NLRP3 acts as a stress integrator that translates mitochondrial injury into IL-1β/IL-18 maturation and pyroptotic signaling (Nakahira et al., 2011; Zhou et al., 2011). General inflammasome studies established mitochondrial ROS and mitochondrial DNA release as upstream mechanisms of NLRP3 activation (Nakahira et al., 2011; Zhou et al., 2011), whereas RA-FLS-specific studies link hypoxia, ROS and mitophagy-related stress to NLRP3-associated pyroptotic signaling (Hong et al., 2022; Hong et al., 2024).

Lysosomal injury and crystal-like stimuli may also contribute to inflammasome activation in the rheumatoid synovium. RA joints contain immune complexes, fibrin deposits, cartilage degradation products, and particulate material that can be internalized by synovial cells. In macrophages, phagocytosed crystals and particles induce lysosomal rupture and cathepsin release, thereby promoting NLRP3 activation. Although FLSs are not professional phagocytes, they can internalize extracellular material and respond to mechanical and matrix-derived stress. Invasive lining FLSs are exposed to fragmented cartilage matrix and altered tissue stiffness, and mechanotransduction may cooperate with inflammatory signaling. These conditions may not fully recapitulate macrophage crystal responses, but they support the broader concept that lysosomal and mechanical stress can reduce the activation threshold for inflammasome signaling in stromal cells (Xiao and Zhang, 2023).

Ion flux provides another mechanistic link between tissue injury and inflammasome activation. Extracellular ATP accumulates in inflamed and damaged tissues. Activation of the P2X7 receptor induces potassium efflux, one of the most extensively characterized triggers of NLRP3 activation (He et al., 2015; Kelley et al., 2019; Koumangoye, 2022). Calcium signaling, chloride flux, and sodium channel-associated membrane alterations may also influence inflammatory signaling. Although the hierarchy of ion channel events in RA-FLSs remains incompletely defined, ATP-rich synovial fluid, cellular necrosis, and mechanical injury provide a biologically plausible setting for ion-mediated inflammasome activation. Direct RA-FLS evidence further shows that pharmacological P2X7R antagonism can suppress oxidative stress and inflammatory mediator production, although this study did not directly establish NLRP3 as the downstream mediator (Liu et al., 2020).

### 4.2 Inflammasome-driven synovial crosstalk and tissue remodeling

The consequences of NLRP3 activation extend beyond FLS death. Mature IL-1β can act on FLSs to increase IL-6, prostaglandin E2, chemokines, and MMPs. IL-18 may influence T-cell and natural killer cell responses and support interferon-γ-related circuits in selected inflammatory contexts. GSDMD pore formation can promote the release of alarmins and DAMPs that activate macrophages and endothelial cells. These mediators establish a self-amplifying inflammatory circuit in which macrophage-derived cytokines prime FLSs, FLS-derived chemokines recruit macrophages and T cells, and inflammasome-derived cytokines further reinforce the same network (Korb et al., 2009; Mousavi et al., 2021). The resulting process involves not only enhanced inflammation but also increased tissue retention of inflammatory cells and progressive synovial remodeling.

NLRP3 signaling also intersects with osteoclastogenesis. RA-associated bone erosion depends on the differentiation and activation of osteoclasts under the influence of RANKL, TNF-α, IL-1β, and IL-17. RA-FLSs contribute RANKL and inflammatory mediators, whereas macrophages and monocytes provide osteoclast precursors (Noss and Brenner, 2008; Pap and Korb-Pap, 2015). In addition to IL-1β, IL-18 provides a more direct stromal link between inflammasome output and osteoclastogenic support. Zhang et al. demonstrated expression of both IL-18 receptor chains in cultured human RA-FLSs and showed that IL-18 increased membrane-bound and soluble RANKL production together with M-CSF and GM-CSF. Although OPG production was also increased, the RANKL/OPG ratio shifted in a direction favoring osteoclast formation and bone resorption (Zhang et al., 2013). Thus, IL-18 may promote osteoclastogenesis indirectly by enhancing the osteoclast-supportive phenotype of RA-FLSs. Conversely, pharmacological NLRP3 inhibition in experimental arthritis reduces inflammatory and joint-damage outcomes (Guo et al., 2018; Li B. Y. et al., 2024). Whether these effects are predominantly macrophage-driven, FLS-driven, or mediated by coordinated interactions between these compartments remains an important unresolved question.

The relationship between NLRP3 signaling and FLS invasiveness is of particular pathophysiological relevance. Invasion requires cytoskeletal remodeling, integrin signaling, focal adhesion turnover, cadherin-11-mediated lining organization, and protease secretion. IL-1β can enhance several of these processes, while NLRP3 activation may also promote invasion indirectly through alarmin release and macrophage activation. Conversely, invasive FLS subsets may generate tissue damage that releases additional DAMPs and extracellular ATP, thereby establishing a reciprocal circuit between tissue invasion and inflammasome activation. This hypothesis could be tested by integrating live-cell imaging, three-dimensional cartilage invasion assays, and pharmacological NLRP3 inhibition in patient-derived FLS systems.

Endothelial and vascular interactions should also be incorporated into an FLS-centered model of NLRP3 signaling. RA synovium is characterized by pathological angiogenesis and an activated endothelial compartment that regulates leukocyte entry, hypoxia-associated remodeling, and vascular permeability (Huo et al., 2025; Zhao et al., 2025). IL-1β, a major downstream effector of NLRP3 activation, together with TNF-α, can induce endothelial adhesion molecules such as ICAM-1, VCAM-1, and E-selectin, thereby facilitating leukocyte adhesion and transendothelial migration (Middleton et al., 2004). In parallel, hypoxia and FLS-derived angiogenic mediators, particularly VEGF-family signals, contribute to remodeling of the synovial vascular niche (Anjiki et al., 2024). Nanomedicine strategies that exploit the enhanced vascular permeability of inflamed synovium, including ELVIS/EPR-like passive targeting and biomimetic delivery systems, may therefore influence both local drug accumulation and inflammasome-associated tissue biology (Li et al., 2021; Xiang et al., 2025). A comprehensive FLS-centered model of NLRP3 signaling therefore includes vascular cells as active components of the inflammatory niche, with functions extending beyond drug distribution.

### 4.3 FLS-specific and spatially resolved validation

Direct evidence for NLRP3 inflammasome activation in RA-FLSs remains heterogeneous and should be interpreted alongside studies indicating predominant myeloid activation in intact synovium. Notably, Guo et al. demonstrated that although NLRP3 inflammasome activity was increased in RA synovial tissue, active caspase-1 localized mainly to infiltrating monocytes/macrophages rather than FLSs (Guo et al., 2018). This finding argues against assuming that bulk synovial NLRP3 activity is stromal in origin and suggests that macrophages may represent the dominant inflammasome-active compartment in at least a subset of RA synovia.

However, this observation does not exclude context-dependent FLS-intrinsic inflammasome activation. Subsequent studies using RA-FLSs under defined inflammatory or stress conditions have demonstrated functional NLRP3-related responses, including caspase-1 activation, IL-1β release and pyroptotic signaling, with genetic or pharmacological perturbation supporting pathway involvement (Liu et al., 2019; Hong et al., 2022; Luo et al., 2022). Taken together, the available evidence is most consistent with a context-dependent model in which macrophages are a major—and potentially predominant—source of NLRP3 activity in intact RA synovium, whereas FLSs retain inducible inflammasome competence under specific inflammatory, hypoxic or stress-related conditions.

Collectively, these findings indicate that NLRP3 signaling in RA-FLSs should be interpreted within a broader synovial network that includes inflammatory priming, mitochondrial and lysosomal stress, ion flux, stromal–immune crosstalk, osteoclastogenesis, and vascular remodeling. Representative evidence linking NLRP3 pathway activation to RA synovial pathology and RA-FLS-related mechanisms is summarized in Table 1.

**TABLE 1:** Evidence hierarchy for NLRP3 signaling in RA and RA-FLSs.

Source/model	Main finding	Evidence interpretation	References
Primary human RA-FLSs	Defined inflammatory or stress conditions induce NLRP3-related inflammatory and pyroptotic responses	Direct, context-dependent FLS evidence; does not establish FLSs as the dominant inflammasome-active population in intact RA synovium	(Nakahira et al., 2011; Zhou et al., 2011; Mathews et al., 2014; Liu et al., 2019; Hong et al., 2022; Luo et al., 2022; Nagy et al., 2022; Sakalyte et al., 2022)
Human RA synovium/synovial fluid	RA joints show increased NLRP3/caspase-1/GSDMD and IL-1β/IL-18 signals, with prominent macrophage localization	Direct human joint evidence, but not FLS-specific; bulk signals cannot define cell of origin	(Mathews et al., 2014; Choulaki et al., 2015; Guo et al., 2018; Zhao et al., 2018; Sakalyte et al., 2022; Zhang et al., 2023b; Li et al., 2025)
Peripheral blood/myeloid cells	RA peripheral blood and myeloid cells show increased NLRP3-related expression or functional inflammasome responses	Human systemic/myeloid evidence; not FLS-intrinsic	(Biniecka et al., 2011; Nakahira et al., 2011; Zhou et al., 2011; Mathews et al., 2014; Choulaki et al., 2015; Bustamante et al., 2017; Weyand and Goronzy, 2017; Dong et al., 2020; Cheng et al., 2021; Li et al., 2024c)
Experimental arthritis models	NLRP3 inhibition reduces inflammation and joint damage in CIA and AIA models	RA-relevant *in vivo* evidence; effects cannot be assigned specifically to FLSs	(Noss and Brenner, 2008; Pap and Korb-Pap, 2015; Guo et al., 2018; Mizoguchi et al., 2018; Croft et al., 2019; Friščić et al., 2021; Li et al., 2024a)
General inflammasome models	mtROS/mtDNA, ion flux and dispersed trans-Golgi structures regulate NLRP3 activation	General mechanistic evidence; not RA-FLS-specific without direct validation	(Nakahira et al., 2011; Zhou et al., 2011; Chen and Chen, 2018)

A major limitation of the current literature is its frequent reliance on macrophages as the principal experimental model for NLRP3 biology. Although this approach is experimentally tractable, it may obscure stromal-cell-specific mechanisms. RA-FLSs differ from macrophages in phagocytic capacity, metabolic configuration, receptor repertoire, epigenetic memory, and tissue residency. Observations derived from bone marrow-derived macrophages should therefore not be assumed to operate identically in primary human RA-FLSs (Pap and Korb-Pap, 2015; Zheng et al., 2024). Future studies should evaluate NLRP3 activation in low-passage RA-FLSs from well-characterized donors, compare lining and sublining subsets where feasible, and incorporate macrophage-FLS co-culture systems, synovial explants, and functional readouts such as invasion, MMP release, RANKL expression, and chemokine-mediated immune-cell recruitment. These endpoints would connect molecular inflammasome activity more directly with RA-relevant tissue behavior.

## 5 The dual role of pyroptosis in RA-FLS biology

### 5.1 Pyroptosis as an amplifier of synovial inflammation

Pyroptosis is generally regarded as a pathogenic form of regulated cell death in inflammatory diseases because it couples cellular lysis with robust release of pro-inflammatory cytokines and DAMPs (Wu et al., 2023). In RA, this interpretation is particularly relevant when pyroptotic signaling occurs within a chronically inflamed synovial microenvironment. Increased expression of inflammasome- and pyroptosis-associated markers in RA synovial tissue and peripheral blood cells supports the involvement of NLRP3 inflammasome activation in active disease (Mathews et al., 2014; Zhang X. et al., 2023). Recent studies have implicated the ROS/GRK2/HIF-1α/NLRP3 axis and HIF-1/BNIP3-mediated mitophagy in the regulation of FLS pyroptosis, indicating that pyroptotic signaling in RA is not confined to professional myeloid inflammasome cells (Hong et al., 2022; Hong et al., 2024). These observations support pyroptosis as an active component of synovial inflammation rather than a secondary consequence of tissue injury.

### 5.2 Context-dependent FLS elimination and therapeutic implications

FLS pyroptosis also raises a mechanistic paradox. RA-FLSs are hyperproliferative and resistant to apoptosis, and impaired apoptosis contributes to synovial hyperplasia, pannus expansion, and persistence of pathogenic stromal cells (Li and Wan, 2013; Zhi et al., 2025). Selective elimination of pathogenic FLSs could theoretically reduce synovial cellular burden and attenuate stromal inflammatory activity. These two therapeutic approaches address different biological objectives and can coexist within a context-dependent framework. The key distinction is between controlled therapeutic elimination and uncontrolled inflammatory pyroptosis. Extensive GSDMD pore formation, IL-1β/IL-18 release, LDH release, and DAMP leakage can further inflame the surrounding synovial tissue. By contrast, selective removal of pathogenic FLSs through apoptosis-like or other non-inflammatory mechanisms may reduce synovial cellular burden without amplifying cytokine release (Li and Wan, 2013; Yue et al., 2025).

This distinction has direct implications for NLRP3-targeted therapy. Suppression of NLRP3-associated pyroptotic signaling may be beneficial when the therapeutic objective is to reduce cytokine maturation and inflammatory amplification (Hong et al., 2022; Mao et al., 2023; Hong et al., 2024). Strategies intended to deplete pathogenic FLSs, however, should minimize inflammatory lysis or be combined with cytokine blockade. Disease stage is also likely to influence therapeutic outcome. During early inflammatory priming, NLRP3 inhibition may prevent establishment of a destructive amplification loop. During established pannus formation, NLRP3 inhibition alone may attenuate inflammation but may not reverse epigenetically stabilized invasive behavior (Mizoguchi et al., 2018; Croft et al., 2019; Smith et al., 2023). Future studies should therefore distinguish early inflammatory, persistent inflammatory, and late tissue-destructive FLS states.

Cellular heterogeneity further complicates interpretation of NLRP3-dependent pyroptotic responses. NLRP3 activation may have distinct consequences across synovial cellular compartments. In sublining inflammatory fibroblasts and lining invasive fibroblasts, its functional relevance should be interpreted in relation to fibroblast subset identity and anatomical localization (Mizoguchi et al., 2018; Croft et al., 2019; Zhang et al., 2019). In macrophages and neutrophils, NLRP3-related outputs may instead reflect canonical myeloid inflammasome biology, whereas endothelial cell involvement should be considered within the vascular inflammatory niche. Bulk synovial measurements of NLRP3, caspase-1, or GSDMD cannot define the cellular source of inflammasome activity. Single-cell transcriptomics, imaging mass cytometry, spatial proteomics, and multiplex immunostaining should therefore be incorporated to identify the FLS subsets in which NLRP3 activity predicts inflammatory persistence, erosive progression, or therapeutic response. Without cellular and spatial resolution, the efficacy of systemic NLRP3 inhibition is likely to appear heterogeneous across patient cohorts. Together, these considerations indicate that the functional outcome of pyroptotic signaling in RA-FLS biology depends on the mode of cell death, disease stage, and cellular context, as summarized in Figure 3.

**FIGURE 3:**
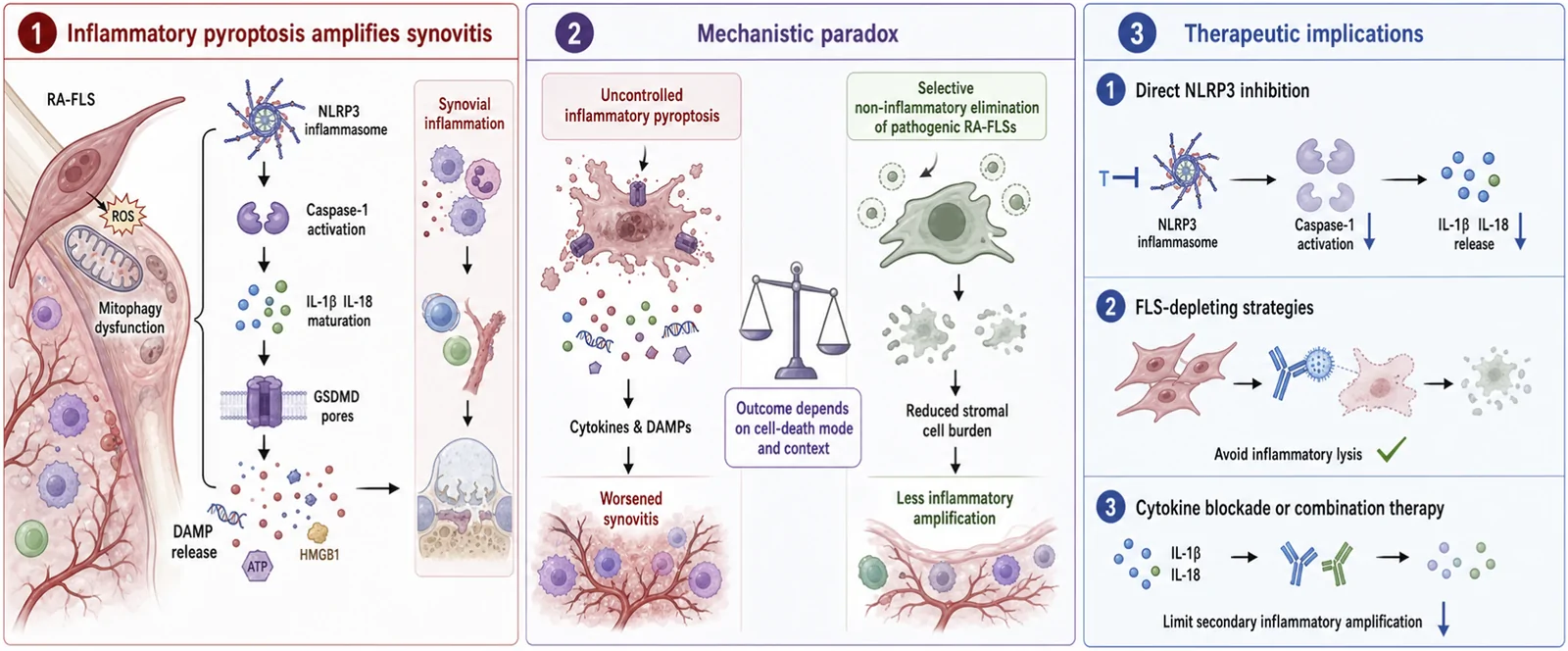
Context-dependent consequences of pyroptosis in RA-FLS biology. In RA-FLSs, mitochondrial stress, mitophagy dysfunction, reactive oxygen species (ROS), and NLRP3 inflammasome activation promote caspase-1 activation, IL-1β/IL-18 maturation, gasdermin D (GSDMD) pore formation, and release of damage-associated molecular patterns (DAMPs), thereby amplifying synovial inflammation. This process creates a mechanistic paradox: uncontrolled inflammatory pyroptosis can aggravate synovitis through cytokine and DAMP release, whereas selective non-inflammatory elimination of pathogenic RA-FLSs may reduce stromal cell burden and limit inflammatory amplification. Therapeutic strategies should therefore distinguish direct NLRP3 inhibition from FLS-depleting approaches and should minimize inflammatory lysis or combine FLS-targeted elimination with cytokine blockade when appropriate. Disease stage, fibroblast subset identity, myeloid cell involvement, endothelial cell context, and spatially resolved validation are critical for interpreting NLRP3-dependent pyroptotic responses in RA synovium. The schematic illustration was created using Affinity Designer.

## 6 Pharmacological targeting of the NLRP3 axis

Pharmacological targeting of the NLRP3 axis in RA should be interpreted according to both the mechanism of intervention and the experimental system in which therapeutic activity has been demonstrated. Numerous compounds have been described as NLRP3 inhibitors or inflammasome modulators on the basis of macrophage assays or studies in inflammatory diseases unrelated to RA; however, such evidence should not be considered equivalent to direct validation in RA synovial cells or experimental arthritis. We therefore prioritize compounds that have been investigated in human RA-FLSs, RA-relevant immune-cell systems, or established experimental arthritis models and explicitly distinguish direct NLRP3 inhibitors from broader pathway modulators. Particular attention is given to whether pharmacological effects were demonstrated directly in RA-FLSs, in myeloid or other immune cells, or only at the whole-animal level. Clinical-stage NLRP3 inhibitors that have not yet been evaluated in RA are discussed separately as translational benchmarks rather than as evidence of RA-specific therapeutic efficacy. Within this evidence-based framework, therapeutic intervention can target inflammasome priming, NLRP3 assembly, upstream stress signals, caspase-1/GSDMD-mediated execution, downstream cytokines, or tissue-specific drug delivery (Figure 4).

**FIGURE 4:**
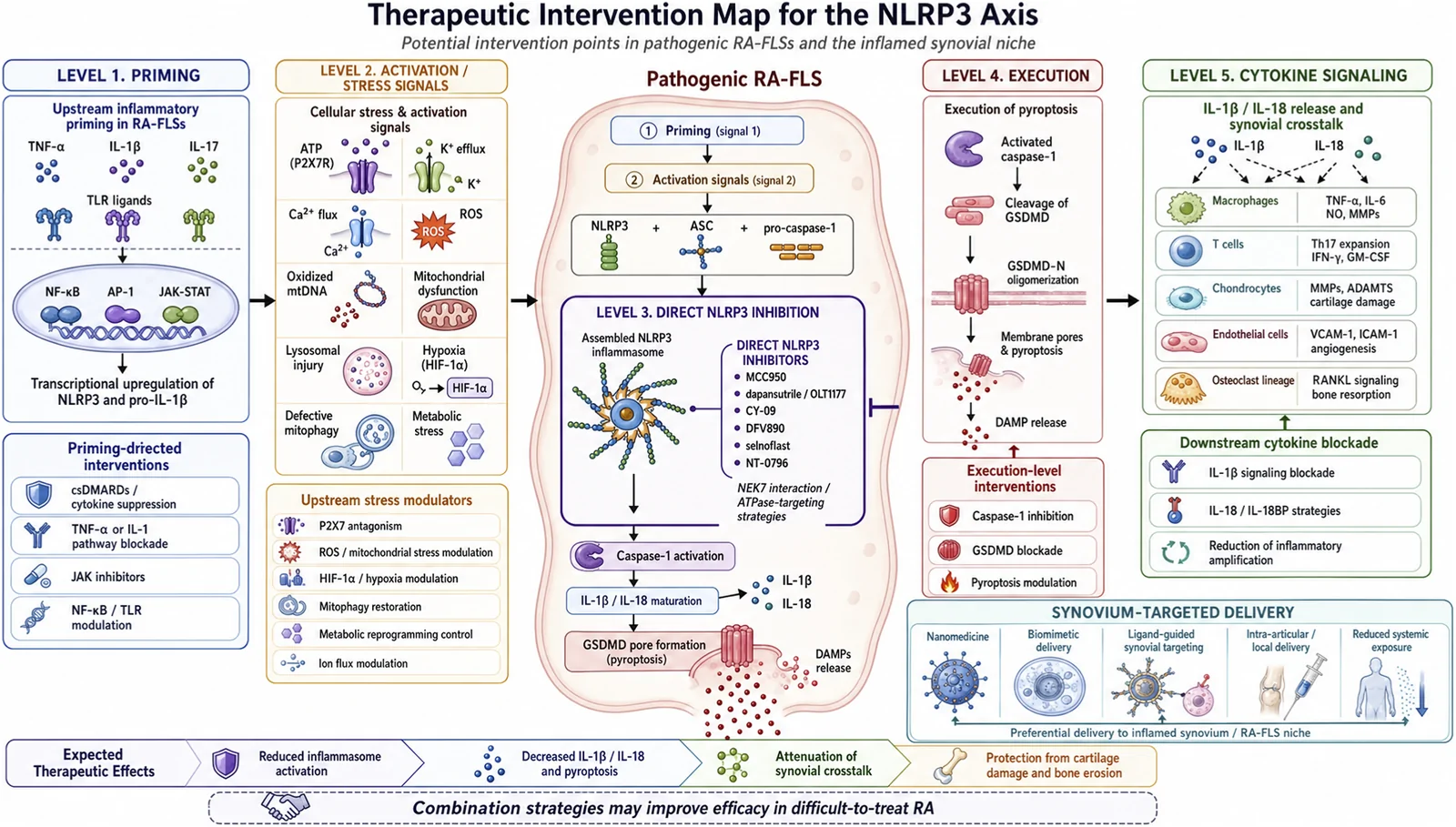
Therapeutic intervention map for the NLRP3 axis. Intervention can occur at the levels of priming, activation, NLRP3 assembly, caspase-1/GSDMD execution and IL-1β/IL-18 signaling. Direct NLRP3 inhibitors should be considered alongside upstream stress modulators, downstream cytokine blockade and synovium-targeted delivery. The schematic illustration was created using Affinity Designer.

### 6.1 Attenuating inflammasome priming with established RA therapies

Priming can be indirectly attenuated by established RA therapies. Methotrexate, TNF inhibitors, IL-6 receptor inhibitors, JAK inhibitors, and glucocorticoids reduce inflammatory transcriptional programs that may limit the availability of NLRP3-related substrates. However, control of upstream priming may be insufficient in synovial tissue in which FLSs remain activated by hypoxia, DAMPs, mechanical stress, and epigenetic memory (Bustamante et al., 2017; Weyand and Goronzy, 2017). This mechanism may contribute to residual synovitis in a subset of patients despite systemic cytokine control. Direct or tissue-targeted inflammasome inhibition could complement current therapies by acting closer to a convergent inflammatory execution node. Among established RA therapies, tofacitinib provides a well-characterized example of indirect NLRP3 modulation in an RA-specific setting. In patients with RA and collagen-induced arthritis (CIA) mice, tofacitinib reduced inflammatory activity and restored the γδTreg/γδT17 balance, while mechanistic experiments in bone-marrow-derived macrophages demonstrated reduced NLRP3 expression, caspase-1 cleavage and IL-1β release. Nlrp3-deficient mice further supported a functional contribution of the inflammasome to the γδT17 response (Yang X. et al., 2021). Importantly, these findings establish myeloid and immune-cell involvement in NLRP3 modulation by tofacitinib but do not demonstrate a direct NLRP3-mediated effect in RA-FLSs.

### 6.2 Direct NLRP3 inhibitors with RA-specific evidence

MCC950 provides the most established pharmacological proof of concept for selective NLRP3 inhibition in experimental RA, although its principal RA-specific efficacy evidence derives from whole-animal and myeloid systems rather than direct treatment of RA-FLSs. In collagen-induced arthritis (CIA) mice, MCC950 significantly reduced clinical arthritis severity, synovial inflammation, cartilage and bone destruction, and synovial IL-1β production (Guo et al., 2018). Importantly, analysis of human RA synovium localized prominent active caspase-1 predominantly to infiltrating monocytes/macrophages rather than fibroblast-like synoviocytes, and the inhibitory effect of MCC950 was further demonstrated in THP-1 monocytic cells (Guo et al., 2018). Thus, MCC950 provides strong RA-relevant *in vivo* and myeloid evidence for NLRP3 inhibition, but the available data should not be interpreted as establishing RA-FLSs as its principal therapeutic target.

More recently, direct NLRP3 inhibitors have been developed specifically in the context of experimental arthritis. Li et al. identified I-19 and the optimized derivative II-8 as small molecules targeting the leucine-rich repeat (LRR) domain of NLRP3 and disrupting the NLRP3–NEK7 interaction. I-19 inhibited caspase-1 activation and IL-1β release and reduced joint swelling and arthritis severity in an adjuvant-induced arthritis (AIA) rat model, while II-8 showed improved inhibitory potency and pharmacokinetic properties (Li B. Y. et al., 2024). These compounds therefore provide RA-relevant *in vivo* evidence for direct molecular targeting of NLRP3; however, their activity has not yet been demonstrated directly in human RA-FLSs.

### 6.3 NLRP3-pathway modulators investigated directly in RA-FLSs

A distinct body of pharmacological evidence has been generated directly in RA-FLS systems. Most compounds in this category are more appropriately classified as NLRP3-pathway modulators because their demonstrated molecular targets lie upstream of NLRP3 or direct physical engagement of NLRP3 has not been established. Nevertheless, these studies are particularly relevant to an FLS-centered therapeutic framework because the pharmacological phenotype and NLRP3-related readouts were measured directly in RA-FLSs rather than inferred from macrophages or whole-joint tissue.

Direct pharmacological perturbation of NLRP3 in RA-FLSs has been demonstrated using INF39. Luo et al. examined FLSs in the context of PLCL1-driven inflammation and showed that PLCL1 overexpression enhanced NLRP3, cleaved caspase-1, IL-1β and other inflammatory outputs in TNF-α-stimulated RA-FLSs. Pharmacological inhibition of NLRP3 with INF39 counteracted the inflammatory effects induced by PLCL1 overexpression, providing direct evidence that inhibition of NLRP3 can suppress inflammatory signaling within the RA-FLS compartment itself (Luo et al., 2022). INF39 was used primarily as a mechanistic NLRP3 probe rather than as a clinically developed RA therapeutic, but the study provides an important distinction from evidence derived exclusively from macrophages or arthritis models.

Gentiopicroside provides direct patient-derived stromal evidence by combining primary human RA-FLS experiments with *in vivo* arthritis validation. Wang et al. used TNF-α-stimulated primary human RA-FLSs and showed that gentiopicroside inhibited FLS proliferation and migration, reduced ROS and NF-κB activation, and suppressed NLRP3, ASC and caspase-1 together with IL-1β production. In parallel, treatment reduced joint swelling, synovial inflammatory infiltration, pannus formation and bone erosion in AIA rats (Wang et al., 2020). These findings provide direct patient-derived RA-FLS pharmacology together with RA-relevant *in vivo* efficacy, although gentiopicroside should be classified as an upstream ROS–NF-κB–NLRP3 pathway modulator rather than a demonstrated direct NLRP3-binding inhibitor.

A monomeric derivative of paeoniflorin (MDP) provides additional mechanistic evidence linking pharmacological intervention to FLS-intrinsic NLRP3 signaling. Hong et al. demonstrated increased HIF-1α, NLRP3, ASC, cleaved caspase-1 and GSDMD-N in RA-FLSs exposed to hypoxic stress. NLRP3 silencing reduced GSDMD-N expression, supporting a causal contribution of NLRP3 to FLS pyroptosis. MDP reduced hypoxia-associated ROS accumulation, IL-1β/IL-18 release and pyroptotic signaling through the ROS/GRK2/HIF-1α/NLRP3 axis, with complementary evidence obtained from adjuvant arthritis models (Hong et al., 2022).

Icariin has also been investigated directly in RA-FLSs through a miR-223-3p/NLRP3 regulatory mechanism. Icariin inhibited RA-FLS proliferation and inflammatory cytokine secretion while promoting apoptosis and increasing miR-223-3p expression. Reporter experiments supported direct targeting of the NLRP3 3′-UTR by miR-223-3p, and inhibition of miR-223-3p reversed the effects of icariin. Importantly, MCC950 restored the anti-inflammatory phenotype in miR-223-3p-deficient RA-FLSs, providing pharmacological rescue evidence for involvement of NLRP3 signaling (Wu et al., 2020).

Several additional compounds reinforce the pharmacological tractability of NLRP3-related pathways in RA-FLS models, although the depth of mechanistic validation varies. Galangin reduced inflammatory mediator production and NF-κB/NLRP3 pathway activation in LPS-stimulated human RA synovial fibroblasts (Fu et al., 2018). Taraxasterol suppressed NLRP3, ASC and caspase-1 together with inflammatory responses in IL-1β-stimulated HFLS-RA cells and also reduced arthritis severity in CIA mice (Chen et al., 2019). Periplogenin inhibited RA-FLS proliferation, migration and invasion and reduced NLRP3/caspase-1/GSDMD-associated pyroptosis and IL-1β/IL-18 release (Ma et al., 2024). Paeoniflorin attenuated RA-FLS inflammation through the hsa_circ_009012/miR-1286/TLR4/NLRP3 axis (Yang et al., 2024), while berberine reduced NLRP3/caspase-1/GSDMD-associated pyroptotic signaling in RA-FLSs and alleviated CIA *in vivo* (Zhang L. et al., 2024). More recently, Butin suppressed proliferation, migration and pyroptotic signaling through the NLRP3/CASP1/GSDMD axis in IL-1β-stimulated HFLS-RA cells (Li et al., 2025). Collectively, these studies support the pharmacological modifiability of NLRP3-related signaling in FLS-based RA models, but reductions in NLRP3 expression or downstream pyroptosis markers alone should not be interpreted as evidence of direct NLRP3 binding.

Celastrol provides a more mechanistically resolved example of indirect NLRP3 modulation. In TNF-α-stimulated FLSs and CIA mice, celastrol reduced FLS proliferation and migration, ROS production, inflammatory cytokine release and NF-κB/NLRP3 signaling. Biophysical and co-immunoprecipitation experiments further demonstrated direct interaction of celastrol with Hsp90 and disruption of the Hsp90–NLRP3 complex. Thus, celastrol has molecular target-engagement evidence for Hsp90 and direct FLS pharmacology, but should not be classified as a direct NLRP3-binding inhibitor (Yang et al., 2025).

Recent work with salvianolic acid B (SalB) further strengthens the upstream-targeting concept. SalB suppressed TNF-α-induced proliferation, migration, invasion and pyroptosis in RA-FLSs and alleviated synovial inflammation and joint damage in AIA rats. Mechanistically, SalB promoted Nrf2 activation, reduced ROS-dependent NLRP3 activation, caspase-1/GSDMD cleavage and IL-1β/IL-18 release. Nrf2 inhibition attenuated these effects, while cellular thermal shift assays, molecular docking and mutational analysis demonstrated direct binding of SalB to Keap1 at Arg415. Thus, SalB provides direct RA-FLS pharmacology and upstream molecular target-engagement evidence, although Keap1 rather than NLRP3 is the demonstrated direct molecular target (Zhou et al., 2026).

Other mechanistically plausible approaches, including P2X7 antagonism, lysosomal stabilization, cathepsin inhibition and generic mitochondrial protection, have substantial support in general inflammasome biology but comparatively limited direct pharmacological validation in human RA-FLSs and are therefore not emphasized here as established RA-FLS therapeutic strategies.

Taken together, the natural-product literature in RA-FLSs should be interpreted at different levels of mechanistic confidence. Celastrol and salvianolic acid B provide molecular target-engagement evidence for upstream regulators, Hsp90 and Keap1, respectively, whereas gentiopicroside, icariin, paeoniflorin derivatives, taraxasterol, periplogenin, paeoniflorin, berberine, Butin and galangin are currently supported primarily as NLRP3-pathway modulators. For the latter compounds, reductions in NLRP3 expression, caspase-1 activation or pyroptotic readouts should not be interpreted as evidence of direct NLRP3 binding.

### 6.4 Targeting downstream inflammasome outputs in RA

Clinical experience with anakinra, canakinumab and rilonacept has established the pharmacological feasibility of IL-1 pathway blockade across RA and other IL-1-mediated inflammatory diseases (Cohen and Rubbert, 2003; Fleischmann et al., 2006; Terkeltaub et al., 2009; Alten et al., 2011; Ilowite et al., 2014). More recent real-world evidence has further supported the effectiveness and acceptable safety profile of anakinra in difficult-to-treat gout, although these observations cannot be directly extrapolated to long-term treatment in RA (Jeria-Navarro et al., 2022). Despite this proof of concept, IL-1 blockade occupies a limited role in current RA management. Contemporary EULAR recommendations prioritize conventional synthetic DMARDs and, when treatment escalation is required, established biological and targeted synthetic DMARDs, including TNF inhibitors, IL-6 receptor inhibitors, B-cell-directed therapy and JAK inhibitors; IL-1 inhibitors are not included among the principal recommended treatment pathways (Smolen et al., 2023).

Selective inhibition of caspase-1 or GSDMD represents a mechanistically attractive downstream strategy, but RA-specific therapeutic evidence remains substantially less developed than that for upstream NLRP3 modulation. In particular, direct pharmacological studies of selective caspase-1 or GSDMD inhibitors in primary human RA-FLSs remain limited. Current RA-FLS studies more commonly demonstrate reductions in caspase-1 cleavage, GSDMD-N formation and pyroptosis as downstream consequences of upstream NLRP3-pathway modulation rather than through direct pharmacological inhibition of these execution proteins (Hong et al., 2022; Ma et al., 2024; Li et al., 2025). At present, caspase-1 and GSDMD serve primarily as mechanistic intervention points and pharmacodynamic readouts; neither is an established therapeutic target in RA.

### 6.5 Clinical-stage NLRP3 inhibitors without RA-specific efficacy data

Several selective NLRP3 inhibitors have progressed into clinical development in inflammatory disorders other than RA. Dapansutrile/OLT1177 has undergone human evaluation in gout and related inflammatory settings (Klück et al., 2020), selnoflast has been investigated clinically in ulcerative colitis (Klughammer et al., 2023), and DFV890 and NT-0796 have provided additional human pharmacological or disease-specific development data outside RA (Gatlik et al., 2024; Clarke et al., 2025). These compounds are important because they demonstrate that selective, orally bioavailable or tissue-penetrant NLRP3 inhibition is feasible in humans. However, none should currently be considered evidence of therapeutic efficacy in RA. Their relevance to the present Review is therefore translational: they provide pharmacokinetic, pharmacodynamic and safety benchmarks for future RA-specific trials rather than RA efficacy evidence.

### 6.6 Synovium- and FLS-targeted delivery strategies

Targeted delivery may be particularly valuable when NLRP3-related activity is restricted to defined synovial cell populations. Recent RA-specific studies have begun to move beyond theoretical nanocarrier design. For example, an FLS-membrane biomimetic liposomal system (FMPlipo@C) was developed to enhance homologous delivery of chrysin to synoviocytes in inflamed joints. In CIA mice, the formulation reduced synovial inflammation, cartilage injury and bone erosion while suppressing the HIF-1α/iNOS/NLRP3 pathway. This study provides RA-specific proof of concept for coupling FLS-oriented delivery with NLRP3-related pathway modulation, although the degree of cell-specific NLRP3 target engagement *in vivo* remains to be established independently (Chen et al., 2025).

Other FLS-directed systems, including HAP-1-functionalized liposomes and FLS-membrane-coated nanoparticles (Liu et al., 2023; Meng et al., 2023), demonstrate that activated synovial fibroblasts can be preferentially targeted in experimental arthritis, although these platforms do not necessarily act through NLRP3. Such studies are therefore relevant primarily to delivery feasibility. For NLRP3-directed therapy, future systems should demonstrate not only joint accumulation and FLS uptake but also cell-resolved suppression of NLRP3 assembly or downstream functional readouts within the targeted FLS population.

### 6.7 Translational requirements: pharmacology and mechanism validation

For direct inhibitors, selectivity is only an initial requirement. RA therapy typically requires months to years of exposure; therefore, candidate compounds must also demonstrate predictable absorption, limited drug–drug interactions, stable pharmacokinetics in patients receiving methotrexate or biologics, and an acceptable therapeutic window between target engagement and toxicity (Fraenkel et al., 2021; Smolen et al., 2023). Hepatotoxicity, off-target pharmacology, and additive immunosuppression should be evaluated early. Therefore, medicinal chemistry programs should integrate biochemical potency with whole-blood assays, primary synovial cell assays, and translational pharmacodynamic markers (Ma, 2023; Smith et al., 2023; Teske et al., 2024). Suppression of IL-1β release after *ex vivo* ATP/nigericin stimulation may provide a systemic pharmacodynamic readout, whereas synovial biopsy or synovial fluid analysis may be required to demonstrate joint-level target engagement (Teske et al., 2024).

Natural products and traditional medicine-derived compounds require particularly careful mechanistic classification. Reductions in NLRP3 protein abundance, caspase-1 cleavage, GSDMD activation or IL-1β/IL-18 release do not by themselves establish direct engagement of NLRP3 and may instead result from modulation of upstream regulatory pathways (Wu et al., 2020; Hong et al., 2022; Yang et al., 2025; Zhou et al., 2026). Accordingly, the term “direct NLRP3 inhibitor” should be reserved for compounds for which biochemical, biophysical or cellular target-engagement evidence supports direct interaction with NLRP3; otherwise, “NLRP3-pathway modulator” is more appropriate.

This distinction is illustrated by the RA-FLS studies discussed above. Celastrol directly interacts with Hsp90 and disrupts the Hsp90–NLRP3 complex, indicating direct engagement of an upstream NLRP3 regulator rather than NLRP3 itself (Yang et al., 2025). Similarly, salvianolic acid B has been shown by cellular thermal shift assay, molecular docking and mutational analysis to bind directly to Keap1, thereby modulating the Keap1–Nrf2/ROS/NLRP3 axis (Zhou et al., 2026). By contrast, mechanistic support for the paeoniflorin derivative MDP and icariin is based on NLRP3 silencing and MCC950 rescue experiments, respectively (Wu et al., 2020; Hong et al., 2022). These approaches strengthen causal attribution to the NLRP3 pathway but do not demonstrate direct physical binding of the natural product to NLRP3. Binding or target-engagement assays, genetic perturbation, rescue experiments and orthogonal functional inflammasome readouts should therefore be used collectively to define the level of mechanistic confidence.

Finally, delivery design should be aligned with the cellular source of pathogenic inflammasome activity. Macrophage-directed systems may be appropriate for myeloid-dominant NLRP3 activity, whereas FLS-directed systems require validated stromal targeting and direct demonstration of FLS-level target engagement. The model-resolved pharmacological evidence supporting these strategies is summarized in Table 2.

**TABLE 2:** Model-resolved pharmacological evidence for targeting the NLRP3 axis in RA.

Agent/intervention	Experimental model(s)	NLRP3 relationship	Evidence interpretation
MCC950	Human RA synovium; CIA mice; THP-1 cells	Direct/selective NLRP3 inhibitor	Strong RA *in vivo*/myeloid evidence; no direct therapeutic validation in RA-FLSs
I-19/II-8	AIA rats	Direct NLRP3 inhibitors targeting LRR domain/NLRP3–NEK7 interaction	RA-relevant animal evidence; no direct human RA-FLS data
INF39	TNF-α-stimulated RA-FLSs	Pharmacological NLRP3 inhibitor/probe	Direct RA-FLS pharmacological evidence; primarily a mechanistic probe
Gentiopicroside	Primary human RA-FLSs; AIA rats	ROS–NF-κB–NLRP3 pathway modulator	Patient-derived RA-FLS + *in vivo* evidence; indirect NLRP3 targeting
MDP	RA-FLSs under hypoxia; adjuvant arthritis models	ROS/GRK2/HIF-1α/NLRP3 modulator	Direct RA-FLS pharmacology with genetic pathway validation
Icariin	RA-FLSs	miR-223-3p/NLRP3 modulator	Direct RA-FLS evidence with pharmacological rescue
Taraxasterol	HFLS-RA; CIA mice	NF-κB/NLRP3 pathway modulator	HFLS-RA + *in vivo* evidence
Periplogenin	RA-FLSs	NLRP3/caspase-1/GSDMD modulator	Direct RA-FLS evidence; direct binding not established
Celastrol	FLSs; CIA mice	Hsp90–NLRP3 complex modulator	FLS + *in vivo* evidence; Hsp90 is the demonstrated direct target
SalB	RA-FLSs; AIA rats	Keap1–Nrf2/ROS/NLRP3 modulator	Direct RA-FLS + *in vivo* + upstream target-engagement evidence
Anakinra	RA clinical studies	Downstream IL-1 receptor blockade	Direct RA clinical evidence; not NLRP3- or FLS-specific

## 7 From inhibitor design to clinical translation

The translational challenge for NLRP3-targeted therapy in RA extends beyond establishing that NLRP3 contributes to arthritic inflammation. Effective treatment will require suppression of pathogenic inflammasome activity in appropriately selected patients, at the relevant tissue site and disease stage, while maintaining an acceptable safety margin. NLRP3 also contributes to host defense, tissue repair, and antimicrobial immunity. Prolonged systemic inhibition may therefore increase infection risk or impair physiological inflammatory responses, particularly when combined with methotrexate, glucocorticoids, bDMARDs, or tsDMARDs. Translational development should focus on three priorities: confirming pathogenic NLRP3 activity in human synovium, demonstrating adequate synovial target engagement, and enriching for patients whose disease biology is likely to depend on inflammasome activation.

### 7.1 Inhibitor design, target engagement, and synovial exposure

Drug design should prioritize selectivity, target engagement, and synovial tissue exposure. Direct NLRP3 inhibitors should avoid broad suppression of other NOD-like receptors, kinases, or metabolic enzymes. Because many inhibitors engage conformational states of the NACHT domain, structural and biophysical assays should be integrated with cellular inflammasome assays to define both binding and functional inhibition (Tengesdal et al., 2024; Vande Walle and Lamkanfi, 2024). For RA, potency in macrophage-based inflammasome assays is insufficient; candidate compounds should also be tested in primary RA-FLSs, macrophage-FLS co-cultures, synovial explants, and arthritis models that capture chronic tissue remodeling. Target engagement should be assessed using markers such as ASC speck formation, caspase-1 activity, GSDMD cleavage, and mature IL-1β/IL-18 release.

Synovial penetration represents a major but often underappreciated determinant of translational success. Plasma pharmacokinetics do not necessarily predict adequate drug concentrations within inflamed synovial tissue or within FLSs embedded in pannus. Intra-articular delivery may provide high local exposure with reduced systemic risk, but repeated injections may be impractical in polyarticular disease. Systemic delivery can reach multiple joints but requires a broader safety window. Nanocarriers and prodrug strategies may help bridge this gap by enriching drug exposure in inflamed tissue and releasing payloads in response to ROS, pH, enzymes, or hypoxia (Park et al., 2020; Yang Y. et al., 2021; Li et al., 2022). Consequently, future research endeavors should quantify drug concentrations in synovial fluid and, optimally, synovial tissue, rather than solely relying on circulating drug levels.

### 7.2 Biomarker-guided patient enrichment and mechanism-aligned endpoints

Biomarker-guided enrichment may reduce the risk of false-negative results in trials of NLRP3-targeted therapy by limiting enrollment to patients with evidence of biologically relevant inflammasome activity. Rather than enrolling patients solely on the basis of conventional clinical disease activity, early trials should prospectively define an enrichment threshold using one or more prespecified molecular or tissue-level criteria (Pitzalis et al., 2020; Humby et al., 2021; Rivellese et al., 2023). Where feasible, these criteria should be supported by evidence of active pathway signaling rather than NLRP3 expression alone. The specific biomarker classes, their degree of NLRP3 specificity and their current validation status in human RA are discussed in detail in Section 8.

Endpoint timing should be aligned with the expected pharmacological effect. Early mechanism-validation intervals should prioritize pharmacokinetic exposure and pharmacodynamic evidence of pathway suppression, ideally within days to weeks of treatment initiation (Klughammer et al., 2023; Gatlik et al., 2024). Clinical inflammatory endpoints, including swollen joint counts, disease-activity scores and imaging-detected synovitis, can subsequently assess whether molecular target engagement translates into reduced inflammatory activity (Rivellese et al., 2023). Structural outcomes related to FLS invasiveness, osteoclastogenesis or bone erosion will require longer follow-up because these effects are unlikely to be captured during short pharmacodynamic studies. Prespecifying this temporal hierarchy would allow inadequate target engagement to be distinguished from genuine biological or clinical non-response.

An early-phase study could, for example, require evidence of persistent active synovitis together with a predefined inflammasome-enriched molecular profile, followed by assessment of early pathway suppression before progression to longer-term clinical or structural endpoints. This staged approach would provide a clearer test of NLRP3 dependency while limiting exposure of biomarker-negative patients to an investigational therapy.

### 7.3 Early-phase and window-of-opportunity trial strategies

A practical early-phase RA trial could initially enroll patients with active synovitis despite methotrexate and at least one bDMARD or tsDMARD, because this population has substantial unmet need and persistent tissue inflammation (Nagy et al., 2021). Enrichment could be based on ultrasound power Doppler activity combined with elevated synovial or blood inflammasome markers. A brief mechanism validation period can assess safety, pharmacokinetics, *ex vivo* target engagement, alterations in IL-1β/IL-18/GSDMD-related markers, and imaging responses. A longer extension phase would be required to determine effects on DAS28, ACR response, physical function, and structural outcomes. This staged design would avoid premature efficacy claims before confirming target engagement and synovial pharmacodynamic activity (Iaquinta et al., 2023). For biomarker-enriched trial design, R4RA and STRAP provide direct clinical precedents (Humby et al., 2021; Rivellese et al., 2023).

A complementary strategy is a window-of-opportunity study in early untreated RA. Patients undergoing diagnostic synovial biopsy could receive an NLRP3 inhibitor for a brief interval before initiation of standard therapy. Paired tissue samples could then be used to measure changes in inflammasome activation, FLS inflammatory programs, and macrophage–FLS interactions. Such trials may not provide immediate clinical efficacy data, but they are highly informative for mechanism validation. They may also clarify whether NLRP3 activity functions as an early driver of disease persistence or as a downstream consequence of established synovial inflammation (Burgers et al., 2019).

### 7.4 Therapeutic positioning and regulatory translation

Regulatory translation will require a clear rationale explaining why NLRP3 inhibition offers added value over existing RA drugs. The strongest argument is not that NLRP3 represents another inflammatory mediator, but that it integrates multiple stress signals that may not be fully addressed by single-cytokine blockade. Clinical development should therefore focus on patients with persistent tissue stress and inflammasome activation despite standard therapy. The value proposition may be strongest in difficult-to-treat RA, as defined by EULAR criteria, or in erosive disease characterized by persistent synovial inflammatory signatures (Humby et al., 2019; Nagy et al., 2021). Management strategy and endpoint selection in this population should also be aligned with EULAR points to consider for difficult-to-treat RA (Nagy et al., 2022). These potential indications remain hypotheses that require prospective clinical validation. Successful clinical translation will therefore require integrated human validation, FLS-relevant models, synovial exposure assessment, biomarker-enriched trial design, and rational therapeutic positioning.

### 7.5 Safety considerations and risk mitigation for NLRP3-targeted therapy

Safety assessment is particularly important for NLRP3-targeted therapy in RA because treatment would probably require prolonged or repeated exposure, often in combination with existing immunomodulatory drugs. NLRP3 contributes to antimicrobial defense, inflammatory host responses and tissue repair; therefore, sustained inhibition raises potential concerns regarding infection susceptibility, vaccine responsiveness and wound healing. However, these risks remain incompletely defined for selective NLRP3 inhibitors, particularly during long-term treatment in patients with RA, and should not be inferred directly from short-term studies in acute inflammatory diseases (Mangan et al., 2018; Swanson et al., 2019; Chen P. K. et al., 2024).

Drug-related safety also depends on molecular selectivity, systemic exposure and concomitant therapy. Candidate inhibitors should be evaluated for hepatotoxicity, off-target pharmacology, hematological effects and clinically relevant drug–drug interactions. These issues are especially important in RA, where NLRP3 inhibitors may be administered together with methotrexate, glucocorticoids, bDMARDs or tsDMARDs. Combination regimens may produce additive immunosuppression, whereas combinations involving JAK inhibitors require particular attention to infection, hematological, cardiovascular and thrombotic risk profiles associated with the background therapy (Fraenkel et al., 2021; Ytterberg et al., 2022; Smolen et al., 2023). Available early-phase studies of selective NLRP3 inhibitors in non-RA indications provide useful short-term safety and pharmacokinetic information, but they do not establish the safety of chronic NLRP3 inhibition in RA (Klück et al., 2020; Klughammer et al., 2023; Gatlik et al., 2024).

Route of administration and tissue targeting may substantially influence the therapeutic window. Systemic oral inhibitors are practical for polyarticular disease but expose multiple tissues to sustained NLRP3 inhibition. Intra-articular or synovium-targeted delivery could reduce systemic exposure, although local toxicity, biodistribution, immunogenicity and manufacturing-related risks require independent evaluation. Future RA trials should therefore incorporate longitudinal monitoring of infections, hepatic and hematological parameters, vaccine responses where appropriate, wound healing and other indicators of innate immune competence. Safety assessment should also account for background immunosuppression, age, comorbidities and cumulative treatment duration. Overall, the clinical feasibility of NLRP3 inhibition in RA will depend not only on pharmacological selectivity but on achieving durable synovial target engagement within an acceptable long-term therapeutic window.

## 8 Biomarkers and patient stratification

### 8.1 Synovial pathotypes and biomarker-guided patient selection

Biomarker development is central to clinical translation of NLRP3-targeted therapy because RA comprises multiple immunopathological endotypes rather than a single uniform disease entity. Histological and molecular studies have identified lymphoid, myeloid, fibroid, and pauci-immune synovial pathotypes, each with distinct cellular composition and therapeutic implications (Dennis et al., 2014; Bykerk, 2023). NLRP3-directed intervention is therefore most likely to benefit patients with inflammasome-enriched myeloid or stromal inflammatory phenotypes rather than those whose disease is primarily driven by alternative immune or structural pathways. Synovial biopsy is increasingly feasible in research settings and can provide direct information on tissue-level inflammation; however, routine clinical implementation remains limited. A clinically practical biomarker strategy will therefore require integration of molecular and genetic readouts already evaluated in human RA with emerging tissue-resolved and pharmacodynamic markers. Importantly, these biomarkers differ substantially in their level of clinical validation and in their specificity for NLRP3 activation, and should not be treated as equivalent indicators of inflammasome activity.

### 8.2 Clinical evidence and validation status of NLRP3- and inflammasome-related biomarkers

Several NLRP3- and inflammasome-related biomarkers have already been evaluated directly in patients with RA, although their biological proximity to NLRP3 activation and degree of clinical validation differ substantially. Peripheral-blood studies provide some of the strongest evidence for systemic inflammasome activation. Mathews et al. reported increased expression of ASC, NLRP3 isoforms and CASP1 in PBMCs from patients with RA, together with increased caspase-1 and IL-18 levels, thereby demonstrating an inflammasome-associated molecular phenotype in human disease (Mathews et al., 2014). Choulaki et al. subsequently showed increased basal NLRP3, ASC and active caspase-1 in whole-blood cells from patients with active RA. Importantly, TLR3-or TLR4-primed cells followed by ATP stimulation released more IL-1β than cells from healthy controls, and this response was suppressed by caspase-1 inhibition, providing functional evidence of enhanced inflammasome competence rather than expression alone (Choulaki et al., 2015). More recently, NLRP3, ASC and CASP1 transcripts in PBMCs and neutrophils were found to be higher in active than inactive RA, with NLRP3 expression positively correlating with CRP, ESR and DAS28 (Cheng et al., 2021).

Joint-level measurements provide complementary evidence and may more closely reflect the pathogenic tissue compartment. In a study comparing patients with RA, osteoarthritis and healthy controls, Zhang et al. demonstrated increased IL-1β and IL-18 in RA synovial fluid and increased NLRP3, caspase-1 and cleaved GSDMD in RA synovial tissue. Synovial-fluid IL-1β and IL-18 were associated with inflammatory and disease-activity measures, whereas multiplex tissue analysis localized prominent NLRP3/caspase-1/GSDMD activation particularly to synovial macrophages (Zhang X. et al., 2023). These observations support synovial-fluid IL-1β/IL-18 and tissue NLRP3–caspase-1–GSDMD signatures as human RA-supported inflammasome/pyroptosis readouts. However, downstream cytokines or caspase-1 activity alone should not be considered fully NLRP3-specific, because they may also arise from other inflammasome pathways. Accordingly, combined detection of an upstream NLRP3 component together with functional downstream readouts such as active caspase-1, cleaved GSDMD and mature IL-1β/IL-18 provides stronger mechanistic evidence than measurement of any individual analyte.

Recent studies have begun to evaluate multianalyte circulating panels rather than isolated inflammasome components. A 2026 serum proteomics study involving 50 patients with RA and 50 healthy controls identified PCSK9 as a pyroptosis-associated biomarker and confirmed increased circulating NLRP3, caspase-1, GSDMD, IL-1β and IL-18. A combined PCSK9–NLRP3–caspase-1–GSDMD panel discriminated RA from healthy controls with an area under the receiver-operating-characteristic curve of 0.930, with 80% sensitivity and 100% specificity (Wang et al., 2026). This represents direct clinical evaluation of an NLRP3/pyroptosis-associated serum biomarker panel, although it should not yet be interpreted as a validated assay of NLRP3 activity because total circulating pathway proteins do not necessarily reflect inflammasome assembly or target engagement and the diagnostic performance requires independent validation. By contrast, CRP, ESR and IL-6 should be regarded as contextual measures of systemic inflammatory activity rather than biomarkers of NLRP3 activation, although they remain useful for assessing clinical associations with NLRP3-related molecular readouts.

### 8.3 NLRP3-pathway genetic variation in disease susceptibility and treatment response

Genetic variation in the NLRP3 inflammasome pathway provides an additional dimension for RA stratification, although the evidence for disease susceptibility and treatment response is not equally consistent. Mathews et al. investigated variants within NLRP3, CARD8 and related inflammasome genes and reported associations with both RA susceptibility and response to anti-TNF therapy. These observations provided early evidence that inherited variation within the NLRP3 inflammasome complex may influence RA biology as well as therapeutic outcome (Mathews et al., 2014). Additional candidate-gene studies have reported associations between NLRP3 polymorphisms, including rs4612666 and rs10754558, and RA susceptibility or disease-related phenotypes in selected populations (Cheng et al., 2021; Awni et al., 2023). However, these associations have not been uniformly reproduced across cohorts, indicating that NLRP3 variants cannot currently be considered established genetic susceptibility markers for RA.

The pharmacogenetic evidence is somewhat more developed, particularly for TNF inhibitors. In the BRAGGSS cohort of 1,278 patients with RA, genetic variants within the NLRP3 inflammasome pathway were associated with EULAR response to anti-TNF therapy, providing an early link between inflammasome genetics and therapeutic stratification (Mathews et al., 2014). This signal was subsequently examined in 538 anti-TNF-naïve Danish patients, in whom carriers of the NLRP3 rs4612666 variant allele had a lower probability of achieving a good or moderate EULAR response; the association was particularly pronounced among current smokers (Sode et al., 2014). A later Danish analysis involving 469 patients again associated rs4612666 with poorer anti-TNF responsiveness (Sode et al., 2018). More recent clinical observations have also linked NLRP3 rs4612666 and CARD8 variants with disease activity, circulating IL-1β and treatment-response phenotypes (Awni et al., 2023). Together, these studies suggest that NLRP3-pathway variants may contribute to interindividual differences in treatment response, although the magnitude and reproducibility of these effects remain uncertain.

Importantly, replication of individual associations does not constitute definitive clinical validation. In an independent study of 581 biologic-naïve patients with RA, Lopez-Rodriguez et al. evaluated previously reported genetic predictors of TNF-inhibitor response, including NLRP3- and CARD8-related variants, but none reproduced the previously reported treatment-response associations (Lopez-Rodriguez et al., 2018). The current evidence therefore supports NLRP3-pathway variation as a biologically plausible and repeatedly investigated genetic biomarker class, but not as an established susceptibility marker or companion diagnostic. Future studies will require larger multi-ancestry cohorts, standardized treatment-response definitions, prospective validation and integration of genotype with molecular measures of inflammasome activity in blood and synovial tissue. Such multimodal approaches may help determine whether germline variation identifies biologically distinct inflammasome-active RA subgroups or merely modifies pathway activity within broader disease endotypes.

### 8.4 Spatially resolved biomarker panels and pharmacodynamic validation

Earlier synovial profiling studies established associations between tissue molecular phenotypes, treatment response and structural progression in RA (Dennis et al., 2014; Humby et al., 2019; Lewis et al., 2019). More recent cell-resolved studies have refined these observations. Multimodal single-cell profiling of more than 314,000 synovial cells identified distinct cell-type abundance phenotypes whose composition was associated with treatment response (Zhang F. et al., 2023). These findings extend earlier bulk-tissue classifications by resolving the cellular states underlying synovial heterogeneity and provide a stronger framework for cell-resolved biomarker development. A biologically informative tissue panel would ideally combine evidence of inflammasome activity with markers that define cellular identity. For example, co-localization of NLRP3-related activity readouts with macrophage markers could identify myeloid-associated inflammasome activity, whereas co-localization with PDPN, THY1, PRG4, VCAM1 or cadherin-11 could help assign such activity to specific FLS compartments (Smith et al., 2023). Importantly, these lineage and stromal markers are not biomarkers of NLRP3 activation themselves; rather, they provide cellular source attribution. Spatially resolved analyses could further determine whether NLRP3-active populations preferentially occupy lining, sublining, perivascular or cartilage-invasive niches and whether their proximity to osteoclast precursors or vascular structures is associated with erosive disease. These multidimensional signatures may ultimately support patient stratification or cell-directed drug delivery, but their predictive value for response to NLRP3-targeted therapy has not yet been prospectively validated in RA (Bykerk, 2023; Iaquinta et al., 2023).

Dynamic pharmacodynamic assessment will be equally important for the rational clinical development of NLRP3 inhibitors. Baseline enrichment biomarkers may help identify patients with an inflammasome-enriched disease phenotype, whereas pharmacodynamic biomarkers are intended to determine whether an intervention engages and suppresses the relevant pathway. Candidate pharmacodynamic panels for NLRP3 inhibitors could incorporate proximal or assembly-related measures, such as ASC speck formation, together with functional downstream readouts including active caspase-1, GSDMD cleavage and mature IL-1β/IL-18 (Chen X. et al., 2024; Tengesdal et al., 2024; Cabral et al., 2025). Several components of such panels are supported by human RA studies, as discussed above, but they have not yet been prospectively validated as pharmacodynamic biomarkers of selective NLRP3 inhibition in RA. Future early-phase trials should therefore incorporate paired baseline and on-treatment measurements in peripheral blood and, where feasible, synovial tissue or synovial fluid. Failure to suppress these molecular readouts despite adequate systemic exposure would suggest insufficient synovial penetration or pathway inhibition, whereas molecular target engagement without improvement in clinical or imaging endpoints would indicate that NLRP3 is unlikely to be a dominant pathogenic driver in that subgroup. Candidate biomarkers and their current evidence status are summarized in Table 3.

**TABLE 3:** Clinical evidence and validation status of NLRP3- and inflammasome-related biomarkers in RA.

Biomarker	Sample	Evidence in human RA	NLRP3 specificity	Current evidence status
NLRP3, ASC, active caspase-1	Peripheral blood cells	Increased in active RA; enhanced functional inflammasome responsiveness demonstrated *ex vivo*	Moderate when combined	Human RA-supported functional readout
Stimulated IL-1β release	Whole-blood cells	Increased after TLR3/TLR4 priming plus ATP in active RA	Moderate	Human RA-supported functional readout
NLRP3/ASC/CASP1 transcripts	PBMCs, neutrophils	Increased in RA and in active vs. inactive disease; NLRP3 associated with CRP, ESR and DAS28	Moderate–low	Clinically associated molecular readout
NLRP3/caspase-1/cleaved GSDMD	Synovial tissue	Increased in RA vs. OA, with prominent signals in synovial macrophages	Moderate–high when combined	Human RA-supported tissue readout
IL-1β/IL-18	Synovial fluid, serum	Increased in RA; synovial levels associated with inflammatory and disease-activity measures	Low individually	Clinically associated; not NLRP3-specific
PCSK9 + NLRP3 + caspase-1 + GSDMD	Serum	Discriminated RA from healthy controls with AUC 0.930 in a 2026 cohort	Moderate	Clinically evaluated; requires external validation
ASC specks	Blood/synovial samples	Strong mechanistic rationale but limited RA-specific clinical evaluation	Inflammasome-related, not uniquely NLRP3-specific	Exploratory candidate
NLRP3 variants	Genomic DNA	Associated with anti-TNF response across several cohorts, but not consistently reproduced in independent validation	High pathway linkage; not an activity readout	Replicated but inconsistently validated

## 9 Combination strategies and therapeutic positioning

### 9.1 Adjunctive combinations with established RA therapies

NLRP3 inhibition is unlikely to displace established disease-modifying strategies for RA in the near term. Its most plausible role is as an adjunctive or precision therapeutic approach for biologically defined patient groups. Combination with methotrexate may attenuate upstream inflammatory priming, whereas NLRP3 inhibition could limit IL-1β/IL-18 maturation and pyroptotic amplification downstream. This remains a mechanistically plausible combination rather than an established therapeutic strategy. In patients with residual synovitis despite partial cytokine control, combination with TNF or IL-6 receptor inhibitors may be considered when synovial inflammation persists despite suppression of dominant systemic cytokine pathways (McInnes et al., 2016). Combination with JAK inhibitors could provide broad transcriptional suppression together with inflammasome modulation (Yang X. et al., 2021), although infection, hematologic, cardiovascular and thrombotic safety signals would require careful prospective assessment (Ytterberg et al., 2022).

### 9.2 Comorbidity-defined opportunities and stage-specific positioning

NLRP3-directed therapy may also have relevance in comorbidity-defined RA phenotypes. Inflammasome signaling has been implicated in atherosclerotic cardiovascular disease, metabolic dysfunction, gout, osteoarthritis, interstitial lung disease, and neuroinflammatory conditions (Ridker et al., 2017; So and Martinon, 2017; Gairola et al., 2024; Liang et al., 2024). Patients with RA frequently develop cardiovascular, metabolic, and pulmonary comorbidities, creating a rationale for therapies that may reduce both synovial inflammation and systemic inflammasome-associated inflammatory burden. Whether such comorbidity-related benefits occur remains a testable translational hypothesis, given the strong tissue- and disease-context dependence of NLRP3 biology.

One therapeutic positioning approach is early intervention in inflammasome-enriched disease. If NLRP3 activation contributes to the establishment of persistent FLS activation, early inhibition may help prevent epigenetic stabilization of destructive stromal programs (Mizoguchi et al., 2018; Croft et al., 2019; Zhang et al., 2019). This concept would require validation in early RA or undifferentiated inflammatory arthritis cohorts with synovial sampling, biomarker-defined enrichment, and long-term structural follow-up. A second strategy is late-stage intervention in difficult-to-treat RA, in which persistent stromal activation may be less dependent on lymphocyte-driven autoimmunity and more reliant on tissue-resident inflammatory circuits. Patients in this setting should be defined according to EULAR criteria, and study design should account for prior inadequate responses to bDMARDs or tsDMARDs (Nagy et al., 2021). These two contexts are likely to require distinct dosing strategies, combination partners, biomarker criteria, and endpoints.

### 9.3 Delivery modality and synovial pathotype-guided selection

The eventual therapeutic position of NLRP3 inhibition may be strongly influenced by delivery modality. A systemic oral NLRP3 inhibitor would be convenient for polyarticular disease, but it would require an excellent chronic safety profile. In contrast, a synovium-targeted nanocarrier or intra-articular formulation may be more suitable for patients with dominant synovitis in selected joints or as a local rescue strategy (Nosrati et al., 2024; Ren et al., 2024; Xiang et al., 2025). Biologic or antibody-based delivery platforms could potentially exploit FLS surface markers; however, fibroblast heterogeneity and marker expression in normal tissues must be carefully addressed (Lee et al., 2007; Kiener et al., 2009). Consequently, the future clinical applicability of NLRP3-targeted therapy in RA will rely not only on the inhibitor molecule *per se* but also on delivery engineering, synovial exposure, and patient selection.

Combination partners should be selected on the basis of mechanistic complementarity. In TNF-driven, NLRP3-high synovitis, TNF blockade combined with NLRP3 inhibition would have a clear mechanistic rationale. In contrast, an IL-6-dominant acute-phase phenotype with limited inflammasome activity may be better addressed by IL-6 receptor blockade than by NLRP3 inhibition (McInnes et al., 2016). For a fibroid synovial pathotype characterized by invasive FLSs but limited myeloid inflammation, local FLS-directed delivery or anti-invasive strategies may be more appropriate (Lee et al., 2007; Kiener et al., 2009). These examples illustrate why synovial pathotyping may become as important as serological classification for the selection of advanced therapies.

### 9.4 Patient-reported outcomes and clinical value

Therapeutic positioning should also incorporate patient-reported outcomes. IL-1β and inflammasome-associated signaling may contribute to fatigue, pain sensitization and systemic sickness behavior through peripheral inflammatory and central neuroimmune mechanisms (Walsh and McWilliams, 2014; Roerink et al., 2017; Omdal, 2020). More recent experimental evidence has directly implicated the microglial P2X7R–NLRP3–IL-1β axis in central sensitization, with pharmacological NLRP3 inhibition attenuating mechanical hyperalgesia and central sensitization-related readouts *in vivo* (Wang et al., 2023). Recent human data from Long COVID further associate NLRP3 inflammasome-related measures with chronic fatigue and broader physio-affective symptom severity, providing contemporary evidence linking inflammasome activity to fatigue-related symptom domains (Zhang et al., 2026). Although these findings are not specific to RA, they strengthen the biological rationale for assessing symptom outcomes alongside conventional measures of synovial inflammation.

Some patients with RA continue to experience pain and fatigue despite reduced swollen joint counts, indicating that inflammatory control and symptom burden are not always concordant (Walsh and McWilliams, 2014; Orbai and Bingham, 2015). If NLRP3 inhibition ameliorates inflammatory pain or systemic symptoms without producing substantial structural improvement, this effect may still be clinically meaningful. Future trials should therefore include validated measures of pain, fatigue, physical function and health-related quality of life alongside inflammatory and structural endpoints, while distinguishing anti-inflammatory effects from analgesic or placebo-related responses (Orbai and Bingham, 2015). Representative combination strategies and their therapeutic rationale are summarized in Table 4.

**TABLE 4:** Combination strategies for future RA trials.

Combination partner	Rationale	Potential target subgroup	Safety issue	References
Methotrexate	Controls upstream inflammatory priming while NLRP3 inhibitor blocks execution	Early or moderate disease with inflammasome-high markers	Hepatic monitoring and additive tolerability	(Fraenkel et al., 2021; Smolen et al., 2023)
TNF inhibitor	Reduces strong FLS priming signal; NLRP3 inhibitor blocks residual pyroptotic loop	Residual synovitis despite TNF blockade	Infection risk with dual immunomodulation	(McInnes et al., 2016; Fraenkel et al., 2021; Smolen et al., 2023)
IL-6R inhibitor	Suppresses systemic inflammation and FLS activation; complements NLRP3 blockade	High CRP/IL-6 phenotype with synovial inflammasome activity	Neutropenia, lipid changes, infection	(McInnes et al., 2016; Fraenkel et al., 2021; Smolen et al., 2023)
JAK inhibitor	Broad cytokine signaling suppression plus inflammasome modulation	Difficult-to-treat RA with multiple cytokine signatures	Cardiovascular, thrombotic and infection warnings	(Fraenkel et al., 2021; Yang et al., 2021a; Ytterberg et al., 2022)
Nanocarrier delivery	Improves joint exposure and reduces systemic NLRP3 blockade	Localized or tissue-dominant synovitis	Biodistribution, immunogenicity and manufacturing control	(Li et al., 2022; Chen et al., 2025; Xiang et al., 2025)

## 10 Discussion

### 10.1 Cellular source attribution and human-relevant models

Several unresolved issues constrain immediate clinical translation of NLRP3-targeted strategies in RA. A central challenge is the relative contribution of FLS-intrinsic NLRP3 activity compared with macrophage-derived inflammasome signaling. Because macrophages are highly efficient inflammasome effector cells, a substantial proportion of synovial NLRP3-associated signals may arise from the myeloid compartment rather than from FLSs (Guo et al., 2018; Kurowska-Stolarska and Alivernini, 2022). This distinction is therapeutically important, because stromal- and myeloid-dominant inflammasome activity may require different delivery systems, pharmacodynamic markers, and combination partners. FLS-restricted genetic perturbation or FLS-directed pharmacological approaches, combined with spatially resolved co-localization and target engagement measurements in defined synovial cell populations, will therefore be required to establish whether pathogenic NLRP3 activity originates from FLSs rather than neighboring myeloid or other stromal cells (Danks et al., 2016; Vickovic et al., 2022; Castillo et al., 2025). Another limitation is the imperfect correspondence between animal models and human RA. Collagen-induced arthritis, collagen antibody-induced arthritis, and TNF-transgenic models remain valuable, but they differ from human disease in adaptive immune architecture, stromal remodeling, tissue chronicity, and treatment history (Chidomere et al., 2023; Kong et al., 2023). Candidate therapies should therefore be validated in human synovial tissue systems, synovial explants, organoids, or joint-on-chip platforms whenever feasible (Zhang X. et al., 2024).

### 10.2 Inflammasome redundancy and parallel inflammatory cell-death pathways

Inflammasome redundancy and parallel inflammatory cell-death pathways may also attenuate the therapeutic effect of selective NLRP3 inhibition. Other inflammasome sensors and non-pyroptotic regulated cell-death programs may contribute to synovial inflammation in a cell- and disease-context-dependent manner (Mangan et al., 2018; Newton et al., 2021; Wu et al., 2023). Ferroptosis is particularly relevant to an FLS-centered view of regulated cell death in RA because it links iron metabolism and lipid peroxidation to the abnormal survival and inflammatory phenotype of synovial fibroblasts. Recent studies have shown that ferroptotic susceptibility in RA-FLSs is highly context dependent. Sulfasalazine induced ferroptotic death of RA-FLSs through PI3K–AKT–ERK1/2 and p53–SLC7A11 signaling, whereas LKB1–AMPK activity protected patient-derived RA-FLSs from ferroptosis by maintaining GPX4 and SLC7A11 expression (Zhao et al., 2024; Lee et al., 2025). Conversely, ferroptosis may also amplify inflammatory functions of RA-FLSs; ferrostatin-1 reduced PAD4-dependent citrullination, MHC-I antigen presentation and downstream inflammatory responses in RA-FLS–T-cell co-culture and experimental arthritis (Zhu et al., 2025). Recent *in vivo* work further suggests potential crosstalk between ferroptotic and NLRP3-associated pathways: in adjuvant-induced arthritis, modulation of the p53/SLC7A11/ALOX15 axis was accompanied by reduced NLRP3 expression (Eltyar et al., 2025). However, because this latter evidence derives from an experimental arthritis model rather than FLS-specific NLRP3 perturbation, direct molecular crosstalk between ferroptosis and NLRP3 in human RA-FLSs remains to be established. Thus, ferroptosis and pyroptosis should be regarded as mechanistically distinct but potentially interacting stress-responsive death programs in the rheumatoid synovium. Compensatory activation of these pathways could limit the efficacy of NLRP3-directed monotherapy, particularly in patients with highly inflammatory or tissue-destructive synovial phenotypes. Network-based biomarkers may help determine whether NLRP3 functions as a dominant pathogenic node or as one component within a broader inflammatory network (Gong et al., 2024).

### 10.3 Methodological standardization in RA-FLS inflammasome research

The field also requires greater methodological standardization. Current studies differ in stimuli, readouts, FLS passage number, culture conditions, and operational definitions of pyroptosis. Primary RA-FLSs can undergo phenotypic drift *in vitro*, and high-passage cells may no longer reflect disease-relevant tissue states. Standardized experimental frameworks should incorporate low-passage primary FLSs, matched OA or non-inflammatory controls where appropriate, macrophage–FLS co-cultures, synovial organoids, multiplex cytokine assays, caspase-1 activity, GSDMD cleavage, ASC speck imaging, LDH release, and functional assays of migration, invasion, and MMP production (Chidomere et al., 2023; Kong et al., 2023; Zhang X. et al., 2024). Without such standardization, cross-study comparison and reproducibility will remain limited.

### 10.4 Integrated translational priorities

Future research should move toward an integrated translational framework rather than isolated pathway description. Priority areas include cell-resolved mapping of NLRP3 activity in human RA synovium across disease stages and treatment histories; mechanistic dissection of how mitochondrial dysfunction, hypoxia, metabolic reprogramming, complement-driven stromal priming (Friščić et al., 2021), and matrix stress converge on NLRP3 in pathogenic FLS subsets; development of synovium-targeted delivery systems capable of achieving adequate local exposure with reduced systemic toxicity; and biomarker-driven early-phase clinical testing that combines molecular target engagement with imaging and clinical endpoints (Bykerk, 2023; Iaquinta et al., 2023; Gong et al., 2024). Progress in these areas would help move NLRP3 targeting from a compelling inflammatory concept toward a realistic therapeutic strategy for selected patients with RA.

### 10.5 Causal validation and systems-guided precision development

A recurring limitation is the overinterpretation of associative findings. Increased expression of NLRP3, caspase-1, or IL-1β in RA synovium does not establish causality, and reduced expression after treatment does not prove direct drug action. Stronger evidence requires loss- and gain-of-function experiments, rescue studies, direct target engagement assays, cell-specific localization, and disease-relevant functional readouts (He et al., 2016a; Zhang X. et al., 2023; Teske et al., 2024). Explicit acknowledgment of this limitation strengthens the review by distinguishing pathway association from therapeutic validation.

Integration with artificial intelligence and systems pharmacology represents an additional future direction. Multi-omics data from synovium, blood, and imaging could be used to identify inflammasome-enriched endotypes and predict rational combination strategies (Gong et al., 2024). Network models may help determine whether NLRP3 functions as a central driver, a convergent amplifier, or a downstream readout in individual patients. Structure-based modeling may further guide the design of allosteric inhibitors with improved selectivity and synovial exposure. These computational approaches cannot replace biological validation, but they can prioritize hypotheses, reduce late-stage trial failure, and align NLRP3-targeted development with the broader movement toward precision medicine in RA (Momtazmanesh et al., 2022; Gong et al., 2024).

## 11 Conclusion

NLRP3 is a compelling but context-dependent therapeutic target in RA. Its biology connects key features of the inflamed synovium, including cytokine priming, mitochondrial stress, ROS, hypoxia, metabolic adaptation, pyroptosis, and stromal-immune crosstalk. FLSs are central to disease persistence and tissue destruction, and NLRP3 signaling may contribute to their capacity to amplify inflammation and interact with macrophages, T cells, chondrocytes, endothelial cells, and osteoclast-lineage cells. The field should nevertheless avoid oversimplification. NLRP3 is not a universal master switch for RA, and systemic inflammasome inhibition without stratification is unlikely to benefit all patients. The most promising path is FLS-informed, synovium-targeted, and biomarker-guided therapy, potentially combined with established DMARDs or biological agents. A successful translational program will require rigorous human tissue validation, precise inhibitor design, delivery strategies that reach the synovial niche, and clinical trials enriched for inflammasome-active disease. Under these conditions, targeting the NLRP3 axis may expand therapeutic options for patients with refractory or difficult-to-treat RA.

## References

[B1] Almutairi K. B. Nossent J. C. Preen D. B. Keen H. I. Inderjeeth C. A. (2021). The prevalence of rheumatoid arthritis: a systematic review of population-based studies. J. Rheumatol. 48 (5), 669–676. 10.3899/jrheum.200367 33060323

[B2] Alten R. Gomez-Reino J. Durez P. Beaulieu A. Sebba A. Krammer G. (2011). Efficacy and safety of the human anti-IL-1β monoclonal antibody canakinumab in rheumatoid arthritis: results of a 12-week, phase II, dose-finding study. BMC Musculoskelet. Disord. 12, 153. 10.1186/1471-2474-12-153 21736751 PMC3152943

[B3] Andreeva L. David L. Rawson S. Shen C. Pasricha T. Pelegrin P. (2021). NLRP3 cages revealed by full-length mouse NLRP3 structure control pathway activation. Cell 184 (26), 6299–6312.e6222. 10.1016/j.cell.2021.11.011 PMC8763037 34861190

[B4] Anjiki K. Hayashi S. Ikuta K. Suda Y. Kamenaga T. Tsubosaka M. (2024). JAK inhibitors inhibit angiogenesis by reducing VEGF production from rheumatoid arthritis-derived fibroblast-like synoviocytes. Clin. Rheumatol. 43 (11), 3525–3536. 10.1007/s10067-024-07142-9 39302595

[B5] Awni A. A. Hamed Z. O. Abdul-Hassan Abbas A. Abdulamir A. S. (2023). Effect of NLRP3 inflammasome genes polymorphism on disease susceptibility and response to TNF-α inhibitors in Iraqi patients with rheumatoid arthritis. Heliyon 9 (6), e16814. 10.1016/j.heliyon.2023.e16814 PMC10275785 37332933

[B6] Biniecka M. Fox E. Gao W. Ng C. T. Veale D. J. Fearon U. (2011). Hypoxia induces mitochondrial mutagenesis and dysfunction in inflammatory arthritis. Arthritis Rheum. 63 (8), 2172–2182. 10.1002/art.30395 21484771

[B7] Buckley C. D. Ospelt C. Gay S. Midwood K. S. (2021). Location, location, location: how the tissue microenvironment affects inflammation in RA. Nat. Rev. Rheumatol. 17 (4), 195–212. 10.1038/s41584-020-00570-2 33526927

[B8] Burgers L. E. Raza K. van der Helm-van Mil A. H. (2019). Window of opportunity in rheumatoid arthritis - definitions and supporting evidence: from old to new perspectives. RMD Open 5 (1), e000870. 10.1136/rmdopen-2018-000870 31168406 PMC6525606

[B9] Bustamante M. F. Garcia-Carbonell R. Whisenant K. D. Guma M. (2017). Fibroblast-like synoviocyte metabolism in the pathogenesis of rheumatoid arthritis. Arthritis Res. Ther. 19 (1), 110. 10.1186/s13075-017-1303-3 28569176 PMC5452638

[B10] Bykerk V. P. (2023). Clinical implications of synovial tissue phenotypes in rheumatoid arthritis. Front. Med. (Lausanne) 10, 1093348. 10.3389/fmed.2023.1093348 PMC11150787 38841268

[B11] Cabral J. E. Wu A. Zhou H. Pham M. A. Lin S. McNulty R. (2025). Targeting the NLRP3 inflammasome for inflammatory disease therapy. Trends Pharmacol. Sci. 46 (6), 503–519. 10.1016/j.tips.2025.04.007 PMC12570976 40374417

[B12] Castillo F. Mackenzie T. A. Domingo E. Iañez I. Robers M. B. Wilkinson J. (2025). Identification of novel inflammasome inhibitors *via* cellular NLRP3 target engagement assays. SLAS Discov. 35, 100258. 10.1016/j.slasd.2025.100258 40789419

[B13] Chen J. Chen Z. J. (2018). PtdIns4P on dispersed trans-Golgi network mediates NLRP3 inflammasome activation. Nature 564 (7734), 71–76. 10.1038/s41586-018-0761-3 30487600 PMC9402428

[B15] Chen J. Wu W. Zhang M. Chen C. (2019). Taraxasterol suppresses inflammation in IL-1β-induced rheumatoid arthritis fibroblast-like synoviocytes and rheumatoid arthritis progression in mice. Int. Immunopharmacol. 70, 274–283. 10.1016/j.intimp.2019.02.029 30851708

[B17] Chen P. K. Tang K. T. Chen D. Y. (2024a). The NLRP3 inflammasome as a pathogenic player showing therapeutic potential in rheumatoid arthritis and its comorbidities: a narrative review. Int. J. Mol. Sci. 25 (1), 626. 10.3390/ijms25010626 PMC10779699 38203796

[B18] Chen X. Zhang P. Zhang Y. Wei M. Tian T. Zhu D. (2024b). The research progression of direct NLRP3 inhibitors to treat inflammatory disorders. Cell Immunol. 397-398, 104810. 10.1016/j.cellimm.2024.104810 38324950

[B19] Chen M. Wang Z. Chen H. Li J. Guo X. Zhou S. (2025). Biomimetic nanoparticles inhibit the HIF-1α/iNOS/NLRP3 pathway to alleviate rheumatoid arthritis. Nano Lett. 25 (10), 3807–3816. 10.1021/acs.nanolett.4c05782 40033154

[B20] Cheng L. Liang X. Qian L. Luo C. Li D. (2021). NLRP3 gene polymorphisms and expression in rheumatoid arthritis. Exp. Ther. Med. 22 (4), 1110. 10.3892/etm.2021.10544 PMC8383757 34504564

[B21] Chidomere C. I. Wahid M. Kemble S. Chadwick C. Thomas R. Hardy R. S. (2023). Bench to bedside: modelling inflammatory arthritis. Discov. Immunol. 2 (1), kyac010. 10.1093/discim/kyac010 PMC10917191 38567064

[B22] Choulaki C. Papadaki G. Repa A. Kampouraki E. Kambas K. Ritis K. (2015). Enhanced activity of NLRP3 inflammasome in peripheral blood cells of patients with active rheumatoid arthritis. Arthritis Res. Ther. 17 (1), 257. 10.1186/s13075-015-0775-2 26385789 PMC4575443

[B23] Clarke N. Thornton P. Reader V. Lindsay N. Digby Z. Mullen B. (2025). Anti-neuroinflammatory and anti-inflammatory effects of the NLRP3 inhibitor NT-0796 in subjects with parkinson's disease. Mov. Disord. 40 (10), 2199–2208. 10.1002/mds.30307 PMC12553988 40792655

[B24] Cohen S. B. Rubbert A. (2003). Bringing the clinical experience with anakinra to the patient. Rheumatol. Oxf. 42 (Suppl. 2), ii36–ii40. 10.1093/rheumatology/keg331 12817094

[B26] Coll R. C. Robertson A. A. Chae J. J. Higgins S. C. Muñoz-Planillo R. Inserra M. C. (2015). A small-molecule inhibitor of the NLRP3 inflammasome for the treatment of inflammatory diseases. Nat. Med. 21 (3), 248–255. 10.1038/nm.3806 25686105 PMC4392179

[B27] Coll R. C. Hill J. R. Day C. J. Zamoshnikova A. Boucher D. Massey N. L. (2019). MCC950 directly targets the NLRP3 ATP-Hydrolysis motif for inflammasome inhibition. Nat. Chem. Biol. 15 (6), 556–559. 10.1038/s41589-019-0277-7 31086327

[B28] Croft A. P. Campos J. Jansen K. Turner J. D. Marshall J. Attar M. (2019). Distinct fibroblast subsets drive inflammation and damage in arthritis. Nature 570 (7760), 246–251. 10.1038/s41586-019-1263-7 31142839 PMC6690841

[B31] Danks L. Komatsu N. Guerrini M. M. Sawa S. Armaka M. Kollias G. (2016). RANKL expressed on synovial fibroblasts is primarily responsible for bone erosions during joint inflammation. Ann. Rheum. Dis. 75 (6), 1187–1195. 10.1136/annrheumdis-2014-207137 26025971

[B32] Dennis G. Jr. Holweg C. T. Kummerfeld S. K. Choy D. F. Setiadi A. F. Hackney J. A. (2014). Synovial phenotypes in rheumatoid arthritis correlate with response to biologic therapeutics. Arthritis Res. Ther. 16 (2), R90. 10.1186/ar4555 25167216 PMC4060385

[B33] Dong X. Zheng Z. Lin P. Fu X. Li F. Jiang J. (2020). ACPAs promote IL-1β production in rheumatoid arthritis by activating the NLRP3 inflammasome. Cell Mol. Immunol. 17 (3), 261–271. 10.1038/s41423-019-0201-9 30911117 PMC7052171

[B34] Eltyar F. S. El-Tanbouly D. M. Zaki H. F. El-Sayed R. M. (2025). Crosstalk between ferroptosis and NLRP3, a possible therapeutic target in experimentally-induced rheumatoid arthritis: role of P2Y12R inhibition in modulating P53/SLC7A11/ALOX15 signaling. Inflammopharmacology 33 (7), 3947–3967. 10.1007/s10787-025-01841-8 PMC12354577 40622464

[B35] Falconer J. Murphy A. N. Young S. P. Clark A. R. Tiziani S. Guma M. (2018). Review: synovial cell metabolism and chronic inflammation in rheumatoid arthritis. Arthritis Rheumatol. 70 (7), 984–999. 10.1002/art.40504 29579371 PMC6019623

[B36] Fearon U. Hanlon M. M. Wade S. M. Fletcher J. M. (2019). Altered metabolic pathways regulate synovial inflammation in rheumatoid arthritis. Clin. Exp. Immunol. 197 (2), 170–180. 10.1111/cei.13228 30357805 PMC6642871

[B37] Fleischmann R. M. Tesser J. Schiff M. H. Schechtman J. Burmester G. R. Bennett R. (2006). Safety of extended treatment with anakinra in patients with rheumatoid arthritis. Ann. Rheum. Dis. 65 (8), 1006–1012. 10.1136/ard.2005.048371 16396977 PMC1798263

[B39] Fraenkel L. Bathon J. M. England B. R. St Clair E. W. Arayssi T. Carandang K. (2021). 2021 American college of rheumatology guideline for the treatment of rheumatoid arthritis. Arthritis Rheumatol. 73 (7), 1108–1123. 10.1002/art.41752 34101376

[B40] Friščić J. Böttcher M. Reinwald C. Bruns H. Wirth B. Popp S. J. (2021). The complement system drives local inflammatory tissue priming by metabolic reprogramming of synovial fibroblasts. Immunity 54 (5), 1002–1021.e1010. 10.1016/j.immuni.2021.03.003 33761330

[B41] Fu Q. Gao Y. Zhao H. Wang Z. Wang J. (2018). Galangin protects human rheumatoid arthritis fibroblast-like synoviocytes *via* suppression of the NF-κB/NLRP3 pathway. Mol. Med. Rep. 18 (4), 3619–3624. 10.3892/mmr.2018.9422 30152847

[B42] Gaidt M. M. Ebert T. S. Chauhan D. Schmidt T. Schmid-Burgk J. L. Rapino F. (2016). Human monocytes engage an alternative inflammasome pathway. Immunity 44 (4), 833–846. 10.1016/j.immuni.2016.01.012 27037191

[B43] Gairola S. Sinha A. Kaundal R. K. (2024). Linking NLRP3 inflammasome and pulmonary fibrosis: mechanistic insights and promising therapeutic avenues. Inflammopharmacology 32 (1), 287–305. 10.1007/s10787-023-01389-5 37991660

[B44] Gatlik E. Mehes B. Voltz E. Sommer U. Tritto E. Lestini G. (2024). First-in-human safety, tolerability, and pharmacokinetic results of DFV890, an oral low-molecular-weight NLRP3 inhibitor. Clin. Transl. Sci. 17 (5), e13789. 10.1111/cts.13789 PMC11101992 38761014

[B45] Gong X. Su L. Huang J. Liu J. Wang Q. Luo X. (2024). An overview of multi-omics technologies in rheumatoid arthritis: applications in biomarker and pathway discovery. Front. Immunol. 15, 1381272. 10.3389/fimmu.2024.1381272 PMC11319186 39139555

[B46] Guo C. Fu R. Wang S. Huang Y. Li X. Zhou M. (2018). NLRP3 inflammasome activation contributes to the pathogenesis of rheumatoid arthritis. Clin. Exp. Immunol. 194 (2), 231–243. 10.1111/cei.13167 30277570 PMC6194337

[B47] Harrison D. Billinton A. Bock M. G. Doedens J. R. Gabel C. A. Holloway M. K. (2023). Discovery of clinical candidate NT-0796, a brain-penetrant and highly potent NLRP3 inflammasome inhibitor for neuroinflammatory disorders. J. Med. Chem. 66 (21), 14897–14911. 10.1021/acs.jmedchem.3c01398 37874905

[B48] He W. T. Wan H. Hu L. Chen P. Wang X. Huang Z. (2015). Gasdermin D is an executor of pyroptosis and required for interleukin-1β secretion. Cell Res. 25 (12), 1285–1298. 10.1038/cr.2015.139 26611636 PMC4670995

[B49] He Y. Hara H. Núñez G. (2016a). Mechanism and regulation of NLRP3 inflammasome activation. Trends Biochem. Sci. 41 (12), 1012–1021. 10.1016/j.tibs.2016.09.002 27669650 PMC5123939

[B50] He Y. Zeng M. Y. Yang D. Motro B. Núñez G. (2016b). NEK7 is an essential mediator of NLRP3 activation downstream of potassium efflux. Nature 530 (7590), 354–357. 10.1038/nature16959 26814970 PMC4810788

[B51] Hong Z. Zhang X. Zhang T. Hu L. Liu R. Wang P. (2022). The ROS/GRK2/HIF-1α/NLRP3 pathway mediates pyroptosis of fibroblast-like synoviocytes and the regulation of monomer derivatives of paeoniflorin. Oxid. Med. Cell Longev. 2022, 4566851. 10.1155/2022/4566851 PMC8817856 35132350

[B52] Hong Z. Wang H. Zhang T. Xu L. Zhai Y. Zhang X. (2024). The HIF-1/BNIP3 pathway mediates mitophagy to inhibit the pyroptosis of fibroblast-like synoviocytes in rheumatoid arthritis. Int. Immunopharmacol. 127, 111378. 10.1016/j.intimp.2023.111378 38141408

[B54] Hu J. Shi M. Tian M. Xie B. Tan Y. Zhou D. (2026). Nanomedicine-driven therapeutic strategies for rheumatoid arthritis-associated depression: mechanisms and pharmacological progress. Pharm. (Basel) 19 (1), 94. 10.3390/ph19010094 PMC12844736 41599694

[B55] Humby F. Lewis M. Ramamoorthi N. Hackney J. A. Barnes M. R. Bombardieri M. (2019). Synovial cellular and molecular signatures stratify clinical response to csDMARD therapy and predict radiographic progression in early rheumatoid arthritis patients. Ann. Rheum. Dis. 78 (6), 761–772. 10.1136/annrheumdis-2018-214539 30878974 PMC6579551

[B56] Humby F. Durez P. Buch M. H. Lewis M. J. Rizvi H. Rivellese F. (2021). Rituximab *versus* tocilizumab in anti-TNF inadequate responder patients with rheumatoid arthritis (R4RA): 16-Week outcomes of a stratified, biopsy-driven, multicentre, open-label, phase 4 randomised controlled trial. Lancet 397 (10271), 305–317. 10.1016/s0140-6736(20)32341-2 PMC7829614 33485455

[B57] Huo X. Peng Y. Li H. Li C. Liao F. Miao C. (2025). The emerging role of vascular endothelial cell-mediated angiogenesis in the imbalance of RA synovial microenvironment and its clinical relevance. Front. Pharmacol. 16, 1481089. 10.3389/fphar.2025.1481089 PMC12006175 40255565

[B58] Iaquinta F. S. Rivellese F. Pitzalis C. (2023). Synovial biopsies for molecular definition of rheumatoid arthritis and treatment response phenotyping: where can we improve? Expert Rev. Mol. Diagn 23 (12), 1071–1076. 10.1080/14737159.2023.2284774 37979075

[B59] Ilowite N. T. Prather K. Lokhnygina Y. Schanberg L. E. Elder M. Milojevic D. (2014). Randomized, double-blind, placebo-controlled trial of the efficacy and safety of rilonacept in the treatment of systemic juvenile idiopathic arthritis. Arthritis Rheumatol. 66 (9), 2570–2579. 10.1002/art.38699 24839206 PMC4314719

[B60] Jeria-Navarro S. Gomez-Gomez A. Park H. S. Calvo-Aranda E. Corominas H. Pou M. A. (2022). Effectiveness and safety of anakinra in gouty arthritis: a case series and review of the literature. Front. Med. (Lausanne) 9, 1089993. 10.3389/fmed.2022.1089993 PMC9877612 36714095

[B61] Karami J. Aslani S. Tahmasebi M. N. Mousavi M. J. Sharafat Vaziri A. Jamshidi A. (2020). Epigenetics in rheumatoid arthritis; fibroblast-like synoviocytes as an emerging paradigm in the pathogenesis of the disease. Immunol. Cell Biol. 98 (3), 171–186. 10.1111/imcb.12311 31856314

[B62] Kayagaki N. Stowe I. B. Lee B. L. O'Rourke K. Anderson K. Warming S. (2015). Caspase-11 cleaves gasdermin D for non-canonical inflammasome signalling. Nature 526 (7575), 666–671. 10.1038/nature15541 26375259

[B63] Kelley N. Jeltema D. Duan Y. He Y. (2019). The NLRP3 inflammasome: an overview of mechanisms of activation and regulation. Int. J. Mol. Sci. 20 (13). 10.3390/ijms20133328 31284572 PMC6651423

[B64] Kiener H. P. Niederreiter B. Lee D. M. Jimenez-Boj E. Smolen J. S. Brenner M. B. (2009). Cadherin 11 promotes invasive behavior of fibroblast-like synoviocytes. Arthritis Rheum. 60 (5), 1305–1310. 10.1002/art.24453 19404963 PMC3764540

[B65] Klingemann L. E. Mikuls T. R. England B. R. (2026). Epidemiology and outcomes of rheumatoid arthritis. Curr. Opin. Rheumatol. 38 (2), 69–75. 10.1097/BOR.0000000000001142 41606817

[B66] Klück V. Jansen T. Janssen M. Comarniceanu A. Efdé M. Tengesdal I. W. (2020). Dapansutrile, an oral selective NLRP3 inflammasome inhibitor, for treatment of gout flares: an open-label, dose-adaptive, proof-of-concept, phase 2a trial. Lancet Rheumatol. 2 (5), e270–e280. 10.1016/s2665-9913(20)30065-5 33005902 PMC7523621

[B67] Klughammer B. Piali L. Nica A. Nagel S. Bailey L. Jochum C. (2023). A randomized, double-blind phase 1b study evaluating the safety, tolerability, pharmacokinetics and pharmacodynamics of the NLRP3 inhibitor selnoflast in patients with moderate to severe active ulcerative colitis. Clin. Transl. Med. 13 (11), e1471. 10.1002/ctm2.1471 PMC10644327 37962000

[B68] Kong J. S. Jeong G. H. Yoo S. A. (2023). The use of animal models in rheumatoid arthritis research. J. Yeungnam Med. Sci. 40 (1), 23–29. 10.12701/jyms.2022.00773 PMC9946911 36411592

[B69] Korb A. Pavenstädt H. Pap T. (2009). Cell death in rheumatoid arthritis. Apoptosis 14 (4), 447–454. 10.1007/s10495-009-0317-y 19199037

[B70] Koumangoye R. (2022). The role of Cl(-) and K(+) efflux in NLRP3 inflammasome and innate immune response activation. Am. J. Physiol. Cell Physiol. 322 (4), C645–c652. 10.1152/ajpcell.00421.2021 35171697

[B71] Kurowska-Stolarska M. Alivernini S. (2022). Synovial tissue macrophages in joint homeostasis, rheumatoid arthritis and disease remission. Nat. Rev. Rheumatol. 18 (7), 384–397. 10.1038/s41584-022-00790-8 35672464

[B72] Lamkanfi M. Dixit V. M. (2014). Mechanisms and functions of inflammasomes. Cell 157 (5), 1013–1022. 10.1016/j.cell.2014.04.007 24855941

[B73] Lee D. M. Kiener H. P. Agarwal S. K. Noss E. H. Watts G. F. Chisaka O. (2007). Cadherin-11 in synovial lining formation and pathology in arthritis. Science 315 (5814), 1006–1010. 10.1126/science.1137306 17255475

[B74] Lee H. R. Yoo S. J. Kim J. Kang S. W. (2025). The LKB1-AMPK signaling axis modulates ferroptosis in fibroblast-like synoviocytes derived from rheumatoid arthritis. Biomedicines 13 (2), 321. 10.3390/biomedicines13020321 40002734 PMC11853117

[B75] Lefèvre S. Knedla A. Tennie C. Kampmann A. Wunrau C. Dinser R. (2009). Synovial fibroblasts spread rheumatoid arthritis to unaffected joints. Nat. Med. 15 (12), 1414–1420. 10.1038/nm.2050 19898488 PMC3678354

[B76] Lewis M. J. Barnes M. R. Blighe K. Goldmann K. Rana S. Hackney J. A. (2019). Molecular portraits of early rheumatoid arthritis identify clinical and treatment response phenotypes. Cell Rep. 28 (9), 2455–2470.e2455. 10.1016/j.celrep.2019.07.091 31461658 PMC6718830

[B78] Li H. Wan A. (2013). Apoptosis of rheumatoid arthritis fibroblast-like synoviocytes: possible roles of nitric oxide and the thioredoxin 1. Mediat. Inflamm. 2013, 953462. 10.1155/2013/953462 23690674 PMC3649754

[B79] Li S. Su J. Cai W. Liu J. X. (2021). Nanomaterials manipulate macrophages for rheumatoid arthritis treatment. Front. Pharmacol. 12, 699245. 10.3389/fphar.2021.699245 34335264 PMC8316763

[B80] Li H. Feng Y. Zheng X. Jia M. Mei Z. Wang Y. (2022). M2-type exosomes nanoparticles for rheumatoid arthritis therapy *via* macrophage re-polarization. J. Control Release 341, 16–30. 10.1016/j.jconrel.2021.11.019 34793917

[B81] Li B. Y. Li P. Wei L. Y. Zou J. Wang Y. H. You Q. D. (2024a). Discovery and development of NLRP3 inhibitors targeting the LRR domain to disrupt NLRP3-NEK7 interaction for the treatment of rheumatoid arthritis. J. Med. Chem. 67 (12), 9869–9895. 10.1021/acs.jmedchem.3c02407 38888047

[B82] Li J. Li W. Zhuang L. (2024b). Natural biomimetic nano-system for drug delivery in the treatment of rheumatoid arthritis: a literature review of the last 5 years. Front. Med. (Lausanne) 11, 1385123. 10.3389/fmed.2024.1385123 PMC11114446 38784236

[B83] Li P. Zhou M. Wang J. Tian J. Zhang L. Wei Y. (2024c). Important role of mitochondrial dysfunction in immune triggering and inflammatory response in rheumatoid arthritis. J. Inflamm. Res. 17, 11631–11657. 10.2147/jir.S499473 PMC11687318 39741752

[B84] Li H. Yang Y. Ma X. Yao X. Ling Y. Ma W. (2025). Butin regulates the NLRP3/CASP-1/GSDMD pyroptosis axis to inhibit proliferation and pyroptosis in human fibroblast-like synoviocytes of rheumatoid arthritis. Sci. Rep. 15 (1), 33090. 10.1038/s41598-025-17239-z 41006493 PMC12475276

[B85] Liang R. Qi X. Cai Q. Niu L. Huang X. Zhang D. (2024). The role of NLRP3 inflammasome in aging and age-related diseases. Immun. Ageing 21 (1), 14. 10.1186/s12979-023-00395-z 38317229 PMC10840156

[B86] Liu Y. Wei W. Wang Y. Wan C. Bai Y. Sun X. (2019). TNF-α/calreticulin dual signaling induced NLRP3 inflammasome activation associated with HuR nucleocytoplasmic shuttling in rheumatoid arthritis. Inflamm. Res. 68 (7), 597–611. 10.1007/s00011-019-01244-w 31119302

[B87] Liu Y. Wu Y. Gu S. Yin Q. Li H. Wang J. (2020). The P2X7 receptor (P2X7R)-specific antagonist A804598 inhibits inflammatory reaction in human fibroblast-like synoviocytes. Am. J. Transl. Res. 12 (1), 45–53. 32051736 PMC7013224

[B88] Liu Y. Rao P. Qian H. Shi Y. Chen S. Lan J. (2023). Regulatory fibroblast-like synoviocytes cell membrane coated nanoparticles: a novel targeted therapy for rheumatoid arthritis. Adv. Sci. (Weinh) 10 (4), e2204998. 10.1002/advs.202204998 36509660 PMC9896074

[B90] Lopez-Rodriguez R. Perez-Pampin E. Marquez A. Blanco F. J. Joven B. Carreira P. (2018). Validation study of genetic biomarkers of response to TNF inhibitors in rheumatoid arthritis. PLoS One 13 (5), e0196793. 10.1371/journal.pone.0196793 29734345 PMC5937760

[B91] Luo S. Li X. F. Yang Y. L. Song B. Wu S. Niu X. N. (2022). PLCL1 regulates fibroblast-like synoviocytes inflammation *via* NLRP3 inflammasomes in rheumatoid arthritis. Adv. Rheumatol. 62 (1), 25. 10.1186/s42358-022-00252-5 35820936

[B92] Ma Q. (2023). Pharmacological inhibition of the NLRP3 inflammasome: structure, molecular activation, and Inhibitor-NLRP3 interaction. Pharmacol. Rev. 75 (3), 487–520. 10.1124/pharmrev.122.000629 36669831 PMC10121800

[B93] Ma X. Yang Y. Li H. Luo Z. Wang Q. Yao X. (2024). Periplogenin inhibits pyroptosis of fibroblastic synoviocytes in rheumatoid arthritis through the NLRP3/Caspase-1/GSDMD signaling pathway. Int. Immunopharmacol. 133, 112041. 10.1016/j.intimp.2024.112041 38636373

[B94] Mahmoud D. E. Kaabachi W. Sassi N. Tarhouni L. Rekik S. Jemmali S. (2022). The synovial fluid fibroblast-like synoviocyte: a long-neglected piece in the puzzle of rheumatoid arthritis pathogenesis. Front. Immunol. 13, 942417. 10.3389/fimmu.2022.942417 35990693 PMC9388825

[B95] Mangan M. S. J. Olhava E. J. Roush W. R. Seidel H. M. Glick G. D. Latz E. (2018). Targeting the NLRP3 inflammasome in inflammatory diseases. Nat. Rev. Drug Discov. 17 (8), 588–606. 10.1038/nrd.2018.97 30026524

[B96] Mao X. Wu W. Nan Y. Sun W. Wang Y. (2023). SMAD2 inhibits pyroptosis of fibroblast-like synoviocytes and secretion of inflammatory factors *via* the TGF-β pathway in rheumatoid arthritis. Arthritis Res. Ther. 25 (1), 144. 10.1186/s13075-023-03136-1 37559090 PMC10410963

[B97] Mathews R. J. Robinson J. I. Battellino M. Wong C. Taylor J. C. Eyre S. (2014). Evidence of NLRP3-inflammasome activation in rheumatoid arthritis (RA); genetic variants within the NLRP3-inflammasome complex in relation to susceptibility to RA and response to anti-TNF treatment. Ann. Rheum. Dis. 73 (6), 1202–1210. 10.1136/annrheumdis-2013-203276 23687262

[B98] McInnes I. B. Buckley C. D. Isaacs J. D. (2016). Cytokines in rheumatoid arthritis - shaping the immunological landscape. Nat. Rev. Rheumatol. 12 (1), 63–68. 10.1038/nrrheum.2015.171 26656659

[B99] Meng S. Song Z. Tang Z. Yang X. Xiao Y. Guo H. (2023). Surface-decorated nanoliposomal leonurine targets activated fibroblast-like synoviocytes for efficient rheumatoid arthritis therapy. Biomater. Sci. 11 (21), 7099–7113. 10.1039/d3bm00911d 37668226

[B101] Middleton J. Americh L. Gayon R. Julien D. Aguilar L. Amalric F. (2004). Endothelial cell phenotypes in the rheumatoid synovium: activated, angiogenic, apoptotic and leaky. Arthritis Res. Ther. 6 (2), 60–72. 10.1186/ar1156 15059266 PMC400438

[B102] Mizoguchi F. Slowikowski K. Wei K. Marshall J. L. Rao D. A. Chang S. K. (2018). Functionally distinct disease-associated fibroblast subsets in rheumatoid arthritis. Nat. Commun. 9 (1), 789. 10.1038/s41467-018-02892-y 29476097 PMC5824882

[B103] Momtazmanesh S. Nowroozi A. Rezaei N. (2022). Artificial intelligence in rheumatoid arthritis: current status and future perspectives: a state-of-the-art review. Rheumatol. Ther. 9 (5), 1249–1304. 10.1007/s40744-022-00475-4 35849321 PMC9510088

[B104] Mousavi M. J. Karami J. Aslani S. Tahmasebi M. N. Vaziri A. S. Jamshidi A. (2021). Transformation of fibroblast-like synoviocytes in rheumatoid arthritis; from a friend to foe. Auto. Immun. Highlights 12 (1), 3. 10.1186/s13317-020-00145-x PMC7863458 33546769

[B105] Nagy G. Roodenrijs N. M. T. Welsing P. M. Kedves M. Hamar A. van der Goes M. C. (2021). EULAR definition of difficult-to-treat rheumatoid arthritis. Ann. Rheum. Dis. 80 (1), 31–35. 10.1136/annrheumdis-2020-217344 33004335 PMC7788062

[B106] Nagy G. Roodenrijs N. M. T. Welsing P. M. J. Kedves M. Hamar A. van der Goes M. C. (2022). EULAR points to consider for the management of difficult-to-treat rheumatoid arthritis. Ann. Rheum. Dis. 81 (1), 20–33. 10.1136/annrheumdis-2021-220973 34407926 PMC8761998

[B107] Nakahira K. Haspel J. A. Rathinam V. A. Lee S. J. Dolinay T. Lam H. C. (2011). Autophagy proteins regulate innate immune responses by inhibiting the release of mitochondrial DNA mediated by the NALP3 inflammasome. Nat. Immunol. 12 (3), 222–230. 10.1038/ni.1980 21151103 PMC3079381

[B108] Newton K. Dixit V. M. Kayagaki N. (2021). Dying cells fan the flames of inflammation. Science 374 (6571), 1076–1080. 10.1126/science.abi5934 34822265

[B109] Nosrati Z. Chen Y. A. Bergamo M. Rodríguez-Rodríguez C. Chan J. Shojania K. (2024). Prodrug nanomedicine for synovium targeted therapy of inflammatory arthritis: insights from animal model and human synovial joint fluid. Adv. Healthc. Mater 13 (30), e2401936. 10.1002/adhm.202401936 39380387 PMC11616258

[B110] Noss E. H. Brenner M. B. (2008). The role and therapeutic implications of fibroblast-like synoviocytes in inflammation and cartilage erosion in rheumatoid arthritis. Immunol. Rev. 223, 252–270. 10.1111/j.1600-065X.2008.00648.x 18613841

[B111] O'Keefe M. E. Dubyak G. R. Abbott D. W. (2024). Post-translational control of NLRP3 inflammasome signaling. J. Biol. Chem. 300 (6), 107386. 10.1016/j.jbc.2024.107386 38763335 PMC11245928

[B112] Omdal R. (2020). The biological basis of chronic fatigue: neuroinflammation and innate immunity. Curr. Opin. Neurol. 33 (3), 391–396. 10.1097/wco.0000000000000817 32304437

[B113] Orbai A. M. Bingham C. O. 3rd (2015). Patient reported outcomes in rheumatoid arthritis clinical trials. Curr. Rheumatol. Rep. 17 (4), 28. 10.1007/s11926-015-0501-8 25854489 PMC4404708

[B115] Pap T. Korb-Pap A. (2015). Cartilage damage in osteoarthritis and rheumatoid arthritis--two unequal siblings. Nat. Rev. Rheumatol. 11 (10), 606–615. 10.1038/nrrheum.2015.95 26195338

[B116] Park J. Y. Kwon S. Kim S. H. Kang Y. J. Khang D. (2020). Triamcinolone-gold nanoparticles repolarize synoviocytes and macrophages in an inflamed synovium. ACS Appl. Mater Interfaces 12 (35), 38936–38949. 10.1021/acsami.0c09842 32805872

[B117] Pitzalis C. Choy E. H. S. Buch M. H. (2020). Transforming clinical trials in rheumatology: towards patient-centric precision medicine. Nat. Rev. Rheumatol. 16 (10), 590–599. 10.1038/s41584-020-0491-4 32887976

[B118] Qian H. Deng C. Chen S. Zhang X. He Y. Lan J. (2024). Targeting pathogenic fibroblast-like synoviocyte subsets in rheumatoid arthritis. Arthritis Res. Ther. 26 (1), 103. 10.1186/s13075-024-03343-4 38783357 PMC11112866

[B120] Ren S. Xu Y. Dong X. Mu Q. Chen X. Yu Y. (2024). Nanotechnology-empowered combination therapy for rheumatoid arthritis: principles, strategies, and challenges. J. Nanobiotechnology 22 (1), 431. 10.1186/s12951-024-02670-7 PMC11265020 39034407

[B121] Ridker P. M. Everett B. M. Thuren T. MacFadyen J. G. Chang W. H. Ballantyne C. (2017). Antiinflammatory therapy with canakinumab for atherosclerotic disease. N. Engl. J. Med. 377 (12), 1119–1131. 10.1056/NEJMoa1707914 28845751

[B122] Rivellese F. Nerviani A. Giorli G. Warren L. Jaworska E. Bombardieri M. (2023). Stratification of biological therapies by pathobiology in biologic-naive patients with rheumatoid arthritis (STRAP and STRAP-EU): two parallel, open-label, biopsy-driven, randomised trials. Lancet Rheumatol. 5 (11), e648–e659. 10.1016/s2665-9913(23)00241-2 38251532

[B123] Roerink M. E. van der Schaaf M. E. Dinarello C. A. Knoop H. van der Meer J. W. (2017). Interleukin-1 as a mediator of fatigue in disease: a narrative review. J. Neuroinflammation 14 (1), 16. 10.1186/s12974-017-0796-7 28109186 PMC5251329

[B124] Sakalyte R. Denkovskij J. Bernotiene E. Stropuviene S. Mikulenaite S. O. Kvederas G. (2022). The expression of inflammasomes NLRP1 and NLRP3, toll-like receptors, and vitamin D receptor in synovial fibroblasts from patients with different types of knee arthritis. Front. Immunol. 12, 767512. 10.3389/fimmu.2021.767512 35126351 PMC8807559

[B125] Sánchez-Fernández A. Skouras D. B. Dinarello C. A. López-Vales R. (2019). OLT1177 (dapansutrile), a selective NLRP3 inflammasome inhibitor, ameliorates experimental autoimmune encephalomyelitis pathogenesis. Front. Immunol. 10, 2578. 10.3389/fimmu.2019.02578 31736980 PMC6839275

[B126] Sharif H. Wang L. Wang W. L. Magupalli V. G. Andreeva L. Qiao Q. (2019). Structural mechanism for NEK7-licensed activation of NLRP3 inflammasome. Nature 570 (7761), 338–343. 10.1038/s41586-019-1295-z 31189953 PMC6774351

[B127] Shi J. Zhao Y. Wang K. Shi X. Wang Y. Huang H. (2015). Cleavage of GSDMD by inflammatory caspases determines pyroptotic cell death. Nature 526 (7575), 660–665. 10.1038/nature15514 26375003

[B128] Shin H. J. Kim I. S. Kim J. K. Jo E. K. (2026). Molecular mechanisms of NLRP3 inflammasome activation. Exp. Mol. Med. 58 (3), 650–663. 10.1038/s12276-026-01656-9 41876821 PMC13049041

[B129] Smith M. H. Gao V. R. Periyakoil P. K. Kochen A. DiCarlo E. F. Goodman S. M. (2023). Drivers of heterogeneity in synovial fibroblasts in rheumatoid arthritis. Nat. Immunol. 24 (7), 1200–1210. 10.1038/s41590-023-01527-9 37277655 PMC10307631

[B130] Smolen J. S. Aletaha D. McInnes I. B. (2016). Rheumatoid arthritis. Lancet 388 (10055), 2023–2038. 10.1016/S0140-6736(16)30173-8 27156434

[B131] Smolen J. S. Landewé R. B. M. Bergstra S. A. Kerschbaumer A. Sepriano A. Aletaha D. (2023). EULAR recommendations for the management of rheumatoid arthritis with synthetic and biological disease-modifying antirheumatic drugs: 2022 update. Ann. Rheum. Dis. 82 (1), 3–18. 10.1136/ard-2022-223356 36357155

[B132] So A. K. Martinon F. (2017). Inflammation in gout: mechanisms and therapeutic targets. Nat. Rev. Rheumatol. 13 (11), 639–647. 10.1038/nrrheum.2017.155 28959043

[B133] Sode J. Vogel U. Bank S. Andersen P. S. Thomsen M. K. Hetland M. L. (2014). Anti-TNF treatment response in rheumatoid arthritis patients is associated with genetic variation in the NLRP3-inflammasome. PLoS One 9 (6), e100361. 10.1371/journal.pone.0100361 24967817 PMC4072633

[B134] Sode J. Vogel U. Bank S. Andersen P. S. Hetland M. L. Locht H. (2018). Confirmation of an IRAK3 polymorphism as a genetic marker predicting response to anti-TNF treatment in rheumatoid arthritis. Pharmacogenomics J. 18 (1), 81–86. 10.1038/tpj.2016.66 27698401

[B135] Swanson K. V. Deng M. Ting J. P. (2019). The NLRP3 inflammasome: molecular activation and regulation to therapeutics. Nat. Rev. Immunol. 19 (8), 477–489. 10.1038/s41577-019-0165-0 31036962 PMC7807242

[B136] Tapia-Abellán A. Angosto-Bazarra D. Martínez-Banaclocha H. de Torre-Minguela C. Cerón-Carrasco J. P. Pérez-Sánchez H. (2019). MCC950 closes the active conformation of NLRP3 to an inactive state. Nat. Chem. Biol. 15 (6), 560–564. 10.1038/s41589-019-0278-6 31086329 PMC7116292

[B137] Tengesdal I. W. Banks M. Dinarello C. A. Marchetti C. (2024). Screening NLRP3 drug candidates in clinical development: lessons from existing and emerging technologies. Front. Immunol. 15, 1422249. 10.3389/fimmu.2024.1422249 39188718 PMC11345644

[B138] Terkeltaub R. Sundy J. S. Schumacher H. R. Murphy F. Bookbinder S. Biedermann S. (2009). The interleukin 1 inhibitor rilonacept in treatment of chronic gouty arthritis: results of a placebo-controlled, monosequence crossover, non-randomised, single-blind pilot study. Ann. Rheum. Dis. 68 (10), 1613–1617. 10.1136/ard.2009.108936 19635719 PMC2732898

[B139] Teske K. A. Corona C. Wilkinson J. Mamott D. Good D. A. Zambrano D. (2024). Interrogating direct NLRP3 engagement and functional inflammasome inhibition using cellular assays. Cell Chem. Biol. 31 (2), 349–360.e346. 10.1016/j.chembiol.2023.09.016 37858335

[B140] Thomas T. P. Goonewardena S. N. Majoros I. J. Kotlyar A. Cao Z. Leroueil P. R. (2011). Folate-targeted nanoparticles show efficacy in the treatment of inflammatory arthritis. Arthritis Rheum. 63 (9), 2671–2680. 10.1002/art.30459 21618461 PMC3168725

[B142] Vande Walle L. Lamkanfi M. (2024). Drugging the NLRP3 inflammasome: from signalling mechanisms to therapeutic targets. Nat. Rev. Drug Discov. 23 (1), 43–66. 10.1038/s41573-023-00822-2 38030687

[B143] Vickovic S. Schapiro D. Carlberg K. Lötstedt B. Larsson L. Hildebrandt F. (2022). Three-dimensional spatial transcriptomics uncovers cell type localizations in the human rheumatoid arthritis synovium. Commun. Biol. 5 (1), 129. 10.1038/s42003-022-03050-3 PMC8837632 35149753

[B144] Walsh D. A. McWilliams D. F. (2014). Mechanisms, impact and management of pain in rheumatoid arthritis. Nat. Rev. Rheumatol. 10 (10), 581–592. 10.1038/nrrheum.2014.64 24861185

[B145] Wang M. Li H. Wang Y. Hao Y. Huang Y. Wang X. (2020). Anti-rheumatic properties of gentiopicroside are associated with suppression of ROS-NF-κB-NLRP3 axis in fibroblast-like synoviocytes and NF-κB pathway in adjuvant-induced arthritis. Front. Pharmacol. 11, 515. 10.3389/fphar.2020.00515 32477105 PMC7232611

[B146] Wang Y. Dong L. Zhang Y. Zhang Y. Qin G. Zhang D. (2023). Activation of the microglial P2X7R/NLRP3 inflammasome mediates central sensitization in a mouse model of medication overuse headache. Front. Mol. Neurosci. 16, 1177171. 10.3389/fnmol.2023.1177171 37377770 PMC10291138

[B147] Wang L. Chen H. J. Wu T. T. Shen X. Y. Gao Y. Y. Yin Y. F. (2026). Proteomics-based identification of the pyroptosis-related biomarker PCSK9 and its association with the pathogenesis of rheumatoid arthritis. Kaohsiung J. Med. Sci. 42, e70183. 10.1002/kjm2.70183 41665155 PMC13399913

[B148] Weyand C. M. Goronzy J. J. (2017). Immunometabolism in early and late stages of rheumatoid arthritis. Nat. Rev. Rheumatol. 13 (5), 291–301. 10.1038/nrrheum.2017.49 28360422 PMC6820517

[B149] Wu Z. M. Luo J. Shi X. D. Zhang S. X. Zhu X. B. Guo J. (2020). Icariin alleviates rheumatoid arthritis *via* regulating miR-223-3p/NLRP3 signalling axis. Autoimmunity 53 (8), 450–458. 10.1080/08916934.2020.1836488 33084415

[B150] Wu D. Li Y. Xu R. (2023). Can pyroptosis be a new target in rheumatoid arthritis treatment? Front. Immunol. 14, 1155606. 10.3389/fimmu.2023.1155606 37426634 PMC10324035

[B151] Xiang Y. Pan Z. Tian D. Zhang L. Dang W. Ye J. (2025). Recent advances in biomimetic drug delivery for rheumatoid arthritis treatment. Int. J. Nanomedicine 20, 14253–14282. 10.2147/ijn.S553826 41341868 PMC12671108

[B152] Xiao Y. Zhang L. (2023). Mechanistic and therapeutic insights into the function of NLRP3 inflammasome in Sterile arthritis. Front. Immunol. 14, 1273174. 10.3389/fimmu.2023.1273174 37954594 PMC10634342

[B153] Xu J. Núñez G. (2023). The NLRP3 inflammasome: activation and regulation. Trends Biochem. Sci. 48 (4), 331–344. 10.1016/j.tibs.2022.10.002 36336552 PMC10023278

[B154] Yang X. Zhan N. Jin Y. Ling H. Xiao C. Xie Z. (2021a). Tofacitinib restores the balance of γδTreg/γδT17 cells in rheumatoid arthritis by inhibiting the NLRP3 inflammasome. Theranostics 11 (3), 1446–1457. 10.7150/thno.47860 PMC7738886 33391544

[B155] Yang Y. Guo L. Wang Z. Liu P. Liu X. Ding J. (2021b). Targeted silver nanoparticles for rheumatoid arthritis therapy *via* macrophage apoptosis and Re-polarization. Biomaterials 264, 120390. 10.1016/j.biomaterials.2020.120390 32980634

[B156] Yang J. Wei Z. Li H. Lv S. Fu Y. Xiao L. (2024). Paeoniflorin inhibits the inflammation of rheumatoid arthritis fibroblast-like synoviocytes by downregulating hsa_circ_009012. Heliyon 10 (9), e30555. 10.1016/j.heliyon.2024.e30555 38726183 PMC11079323

[B157] Yang J. He B. Dang L. Liu J. Liu G. Zhao Y. (2025). Celastrol regulates the Hsp90-NLRP3 interaction to alleviate rheumatoid arthritis. Inflammation 48 (1), 346–360. 10.1007/s10753-024-02060-z 38874810

[B158] Yin H. Liu N. Sigdel K. R. Duan L. (2022). Role of NLRP3 inflammasome in rheumatoid arthritis. Front. Immunol. 13, 931690. 10.3389/fimmu.2022.931690 35833125 PMC9271572

[B160] Ytterberg S. R. Bhatt D. L. Mikuls T. R. Koch G. G. Fleischmann R. Rivas J. L. (2022). Cardiovascular and cancer risk with tofacitinib in rheumatoid arthritis. N. Engl. J. Med. 386 (4), 316–326. 10.1056/NEJMoa2109927 35081280

[B161] Yue S. Fan J. Xie D. Cao C. Wang Z. Huang J. (2025). Unveiling the therapeutic potential: targeting fibroblast-like synoviocytes in rheumatoid arthritis. Expert Rev. Mol. Med. 27, e18. 10.1017/erm.2025.11 PMC12201960 40468839

[B162] Zhang W. Cong X. L. Qin Y. H. He Z. W. He D. Y. Dai S. M. (2013). IL-18 upregulates the production of key regulators of osteoclastogenesis from fibroblast-like synoviocytes in rheumatoid arthritis. Inflammation 36 (1), 103–109. 10.1007/s10753-012-9524-8 22945280

[B163] Zhang F. Wei K. Slowikowski K. Fonseka C. Y. Rao D. A. Kelly S. (2019). Defining inflammatory cell states in rheumatoid arthritis joint synovial tissues by integrating single-cell transcriptomics and mass cytometry. Nat. Immunol. 20 (7), 928–942. 10.1038/s41590-019-0378-1 31061532 PMC6602051

[B164] Zhang F. Jonsson A. H. Nathan A. Millard N. Curtis M. Xiao Q. (2023a). Deconstruction of rheumatoid arthritis synovium defines inflammatory subtypes. Nature 623 (7987), 616–624. 10.1038/s41586-023-06708-y PMC10651487 37938773

[B165] Zhang X. Wang Q. Cao G. Luo M. Hou H. Yue C. (2023b). Pyroptosis by NLRP3/caspase-1/gasdermin-D pathway in synovial tissues of rheumatoid arthritis patients. J. Cell Mol. Med. 27 (16), 2448–2456. 10.1111/jcmm.17834 PMC10424297 37386795

[B166] Zhang L. Tan M. Mao J. Zhang J. Wang X. Y. Zhang Y. (2024a). Natural compound library screening to identify berberine as a treatment for rheumatoid arthritis. Clin. Rheumatol. 43 (3), 959–969. 10.1007/s10067-024-06871-1 38305937

[B167] Zhang X. Su R. Wang H. Wu R. Fan Y. Bin Z. (2024b). The promise of synovial Joint-on-a-Chip in rheumatoid arthritis. Front. Immunol. 15, 1408501. 10.3389/fimmu.2024.1408501 PMC11422143 39324139

[B168] Zhang Y. Al-Hakeim H. K. Al-Jassas H. K. Maes M. (2026). The NLRP3 inflammasome as a key pathway in the affective and chronic fatigue symptoms of long COVID. J. Transl. Med. 24 (1), 166. 10.1186/s12967-026-07703-3 41535956 PMC12888595

[B169] Zhao C. Gu Y. Zeng X. Wang J. (2018). NLRP3 inflammasome regulates Th17 differentiation in rheumatoid arthritis. Clin. Immunol. 197, 154–160. 10.1016/j.clim.2018.09.007 30236770

[B170] Zhao C. Yu Y. Yin G. Xu C. Wang J. Wang L. (2024). Sulfasalazine promotes ferroptosis through AKT-ERK1/2 and P53-SLC7A11 in rheumatoid arthritis. Inflammopharmacology 32 (2), 1277–1294. 10.1007/s10787-024-01439-6 38407703 PMC11006818

[B171] Zhao F. Hu Z. Li G. Liu M. Huang Q. Ai K. (2025). Angiogenesis in rheumatoid arthritis: pathological characterization, pathogenic mechanisms, and nano-targeted therapeutic strategies. Bioact. Mater 50, 603–639. 10.1016/j.bioactmat.2025.04.026 40453697 PMC12124647

[B172] Zheng Y. Wei K. Jiang P. Zhao J. Shan Y. Shi Y. (2024). Macrophage polarization in rheumatoid arthritis: signaling pathways, metabolic reprogramming, and crosstalk with synovial fibroblasts. Front. Immunol. 15, 1394108. 10.3389/fimmu.2024.1394108 38799455 PMC11116671

[B173] Zhi X. Zhu H. Sun X. Wang Z. Wang L. Song G. (2025). Dysregulation of different modes of programmed cell death in rheumatoid arthritis fibroblast-like synoviocyte. Int. J. Rheum. Dis. 28 (10), e70445. 10.1111/1756-185x.70445 41128076 PMC12548005

[B174] Zhou R. Yazdi A. S. Menu P. Tschopp J. (2011). A role for mitochondria in NLRP3 inflammasome activation. Nature 469 (7329), 221–225. 10.1038/nature09663 21124315

[B175] Zhou Q. Li T. Fang G. Pang Y. Wang X. (2023). Bioactive molecules against rheumatoid arthritis by suppressing pyroptosis. Pharm. (Basel) 16 (7), 952. 10.3390/ph16070952 PMC10383892 37513864

[B176] Zhou M. Y. Gao Z. Y. Long W. C. Zhou J. Zhang M. Gong X. N. (2026). Salvianolic acid B alleviates rheumatoid arthritis by inhibiting oxidative stress and pyroptosis through the Keap1-Nrf2/ROS/NLRP3 axis. Phytomedicine 150, 157736. 10.1016/j.phymed.2025.157736 41456526

[B177] Zhu X. Lu H. Jia H. Wei X. Xue J. Li W. (2025). Ferrostatin-1 reduces the inflammatory response of rheumatoid arthritis by decreasing the antigen presenting function of fibroblast-like synoviocytes. J. Transl. Med. 23 (1), 280. 10.1186/s12967-025-06300-0 40050869 PMC11884008

